# Primary BPH Stromal Cell Cultures Exhibit Heightened Androgen‐Responsiveness

**DOI:** 10.1002/pros.70239

**Published:** 2026-08-30

**Authors:** Wei Chen, Fulu Dong, Mohammad Reza Askarpour, Laura E. Pascal, Rajiv Dhir, Uma Chandran, Bruce L. Jacobs, Donald B. DeFranco, Naoki Yoshimura, Zhou Wang

**Affiliations:** ^1^ Department of Urology University of Pittsburgh School of Medicine Pittsburgh Pennsylvania USA; ^2^ Department of Pharmacology and Chemical Biology University of Pittsburgh School of Medicine Pittsburgh Pennsylvania USA; ^3^ UPMC Hillman Cancer Center University of Pittsburgh School of Medicine Pittsburgh Pennsylvania USA; ^4^ Department of Pathology University of Pittsburgh School of Medicine Pittsburgh Pennsylvania USA; ^5^ Department of Biomedical Informatics University of Pittsburgh School of Medicine Pittsburgh Pennsylvania USA

**Keywords:** 3D culture, androgen action, benign prostatic hyperplasia (BPH), prostate, stromal‐epithelial interaction

## Abstract

**Background:**

Benign prostatic hyperplasia (BPH) is the most common disease in aging males. BPH growth is androgen‐dependent, and it appears that androgens preferentially stimulate the growth of BPH as compared to normal prostate. The mechanism of preferential androgen stimulation of BPH growth is not clear. Our previous studies suggest that primary BPH stromal cells, but not the paired normal adjacent prostate (NAP) stromal cells from the same patients, could stimulate prostatic epithelial cell proliferation in 3D co‐culture. However, whether androgen regulation of prostatic stromal cells is altered in BPH nodules is not clear.

**Methods:**

Paired BPH and NAP primary stromal cells were prepared from the same patients. Early passage paired BPH and NAP primary cells were treated with androgens and/or antiandrogens for RNA‐seq analysis. RNA‐seq data were analyzed using principal component analysis (PCA) and functional enrichment analysis. Conditioned medium of the paired BPH and NAP primary cells in the presence or absence of androgens were used in three‐dimensional (3D) culture of benign prostatic epithelial cells.

**Results:**

RNA‐seq data analysis suggests that androgen‐induced gene expression in BPH primary stromal cells is heightened compared to paired NAP stromal cells. AR activation in BPH stromal cells appears to enhance cellular motility, vascular and extracellular matrix remodeling, and paracrine signaling, while simultaneously modulating cell cycle and metabolic processes. Compared to regular BPH stromal conditioned medium, conditioned medium of androgen‐stimulated BPH stromal cells further enhanced prostatic epithelial cell proliferation in 3D culture. In contrast, medium of NAP stromal cell culture conditioned in the presence of androgen had no significant effect on prostatic epithelial cell proliferation in 3D culture.

**Conclusions:**

Our findings suggest that androgen stimulation of prostatic epithelial cell growth via paracrine prostatic stromal signaling is enhanced in BPH and is associated with heightened androgen‐responsiveness of BPH stromal cells.

## 1 Introduction

Benign prostatic hyperplasia (BPH) is one of the most common diseases in old men and is a major cause of lower urinary tract symptoms (LUTS) that can significantly impact quality of life. Aging and androgens are two well‐established risk factors for BPH/LUTS [1]. BPH symptoms become more prevalent with age, with one quarter of men in their 50 s, one third of men in their 60 s, and about half of all men 80 years or older presenting with LUTS of BPH [2]. The importance of androgens, particularly DHT, in BPH development and progression is underscored by the lack of reported BPH in males with 5α‐reductase mutations and the efficacy of 5α‐reductase inhibitors, finasteride and dutasteride, in reducing LUTS associated with BPH [3, 4]. In addition, inflammatory factors [5, 6, 7], estrogens [8], non‐steroidal testicular factors [9], and anti‐apoptotic factors [10] have been postulated to contribute to BPH development. However, the mechanisms by which these factors influence BPH/LUTS are poorly understood.

In 1978, McNeal proposed the embryonic reawakening hypothesis to explain BPH development; a reawakening of inductive stroma within the prostate transition‐periurethral zone (TPZ) induces benign neoplastic growth in the aging prostate [11, 12, 13]. This hypothesis was later supported by studies of Cunha et al., showing the importance of stromal‐epithelial interactions in prostate development [14]. Implanting embryonic urogenital sinus mesenchyme (UGM) with non‐glandular adult urinary bladder epithelium in adult male hosts for 4 weeks resulted in fully differentiated prostatic tissue recombinants, indicating the potential of UGM to induce prostatic morphogenesis and differentiation in the presence of male hormones. Additionally, knockout of AR in the murine prostate stromal cell reduced epithelial development in vivo [15], further substantiating a critical role of stromal AR in prostate development and regulation of epithelial proliferation. These findings suggest that stromal AR plays an essential role in prostatic epithelial cell proliferation in BPH progression. An elegant study showed that recombining primary BPH stromal cells with BPH‐1 epithelial cells resulted in small, well‐organized grafts, demonstrating a moderate inductive potential of BPH stromal cells [16]. However, the details of androgen‐dependent paracrine signaling from BPH stromal cells in stimulating prostatic epithelial cell proliferation are still lacking, and the molecular and cellular mechanisms of embryonic reawakening remain unclear.

In our previous study, we isolated and characterized primary stromal cells from patients with BPH nodules [17]. Primary stromal cells derived from BPH patients expressed increased vimentin levels compared to normal adjacent prostate (NAP), suggesting an increased prevalence of fibroblasts and myofibroblasts. Additionally, BPH stromal cells or conditioned medium from BPH stromal cells stimulated prostatic epithelial cell growth, while NAP stromal cells exhibited little or no effect on epithelial growth. These findings suggest that unlike primary NAP stromal cells, primary BPH stromal cells are capable of stimulating prostatic epithelial cell proliferation via paracrine pathways. However, it is not clear if androgens regulate the paracrine factors from primary BPH stromal cells and if primary NAP stromal cells can also mediate androgen stimulation of prostatic epithelial cell proliferation.

Here, RNA‐Sequencing was utilized to identify the androgen‐response genes in primary BPH and NAP stromal cells. We also used 3D heterotypic human prostate culture to investigate the effect of androgens on stimulation of the growth of prostatic epithelial cells by primary BPH versus NAP stromal cells. The findings provided evidence for functional differences between BPH and NAP stromal cells.

## 2 Materials and Methods

### 2.1 Prostate Tissue Collection and Processing

Residual prostate tissue specimens used in this study included paraffin‐embedded tissue blocks and fresh specimens obtained from patients with low‐volume prostate cancer and BPH undergoing prostatectomy at the University of Pittsburgh Medical Center, under an approved Institutional Review Board protocol through the Pitt Biospecimen Core at the University of Pittsburgh. Fresh prostatic tissue specimens were dissected under sterile conditions and histologically classified by a board‐certified genitourinary pathologist (R. Dhir) immediately after surgery. Areas of BPH and NAP from the transition zone and/or central zone of the prostate were confirmed by examining frozen sections of tissues procured adjacent to the sample submitted for clinical histologic assessment. Normal adjacent tissues were without any histopathological changes. Thereafter, the tissue samples were transported to the cell culture laboratory in 50/50 Dulbecco's modified Eagles medium (DMEM)/F12 (10‐090‐CV, Corning Inc., Corning, NY, USA), supplemented with 1 µg/mL insulin‐transferrin‐selenium‐X (51500056, Invitrogen, Waltham, MA, USA), 0.4% bovine pituitary extract (13028014, Gibco, Waltham, Massachusetts, USA), 1% penicillin and streptomycin, and 3 ng/mL epidermal growth factor (S0155, Gibco).

### 2.2 Primary Stromal Cell Culture

BPH and NAP tissue specimens were minced and dissociated by incubation in 2.4 U/ml Dispase II (04942078001, Roche Applied Science, Penzberg, Germany) at 37°C for 1 h on a rocker to generate a suspension [17]. A volume of 1 ml cell suspension was diluted with 4 mL of 50/50 Dulbecco's modified Eagles medium (DMEM)/F12 included 1 µg/mL insulin‐transferrin‐selenium‐X, 0.4% bovine pituitary extract, 1% penicillin and streptomycin, and 3 ng/mL epidermal growth factor in 6 cm dishes. Plated cells were incubated at 37°C, 5% CO_2_ for 5 days without disturbing. On day five, 2 mL of fresh medium was added to each dish to compensate for evaporation. Stromal cell proliferation was typically evident by day seven. When stromal cells reached 95% confluence, cells were serially passaged using a trypsin:EDTA (0.25%:0.53 mM) solution and neutralized with medium. Primary stromal cell identity was confirmed as previously described using established stromal markers [17]. Cultures were routinely monitored and consistently exhibited the characteristic spindle‐shaped morphology of prostate stromal cells, with no apparent epithelial‐like colonies observed during culture expansion. Primary cells with 5 or fewer passages were used in RNA sequencing, Western blotting, and co‐culture experiments.

### 2.3 Western Blotting

Primary cultures of BPH and NAP cells were collected for Western blot assay. RIPA lysate solution was used to prepare cell lysates, and the protein concentration was measured using a BCA Protein Assay Kit (23225, Thermo Fisher Scientific, Waltham, MA, USA). After Western transfer, the membranes were blocked and then incubated with primary antibodies AR (Cell Signaling Technology, ab‐46794, 1: 1000 dilution) and GAPDH (Santa Cruz Biotechnology, sc‐47724, 1:3000 dilution). Subsequently, the membranes were incubated with corresponding secondary antibodies and assessed with chemiluminescence. Quantification of AR protein levels was performed using ImageJ software and normalized to GAPDH.

### 2.4 RNA Sequencing

Primary stromal cells derived from BPH or NAP were seeded into 6 cm dishes and grown in medium with 1 nM R1881 or 1 nM R1881 plus 10 μM MDV3100 for 2 days to reach 100% confluence prior to collection. Total RNA was extracted from stromal cells using RNeasy Mini Kit (Qiagen, Cat No. 74104, Hilden, Germany). Three patient‐derived stromal cell cultures with robust growth, viability, and cellular morphology were selected for transcriptomic analysis. Sequencing was carried out by Novogene (Sacramento, CA, USA).

### 2.5 RNA‐Seq Data Processing and Differential Expression Analysis

The RNA seq data can be accessed in GEO with id GSE344492. Transcript abundance was quantified in terms of transcripts per million (TPM) using kallisto v0.46.0 [18], with an index constructed from the Ensembl GRCh38 reference transcriptome. The resulting TPM values for each sample were then compiled into a transcript expression matrix for subsequent analyses. tximport (1.30.0) was employed to convert the kallisto output into raw counts, which were subsequently analyzed for differential expression using *edgeR* (version 4.0.16). Differentially expressed genes were selected based on a log fold change (logFC) ≥ 1 and *p*‐value < 0.05 for upregulated genes, and logFC ≤ −1 and *p*‐value < 0.05 for downregulated genes.

### 2.6 Principal Component Analysis (PCA) and Functional Enrichment Analysis

PCA was performed using the FactoMineR package (version 2.11) in R, and the results were visualized with the ggord package (version 1.1.7). For functional enrichment analysis, KEGG pathway enrichment analysis was conducted using the clusterProfiler package (version 4.10.1) after mapping gene symbols to ENTREZ Gene IDs to identify significantly enriched biological pathways. Additionally, Gene Ontology (GO) enrichment was performed using g:Profiler, and gene sets with similar functions were clustered using EnrichmentMap.

### 2.7 Three‐Dimensional Culture of Prostatic Epithelial Cells in the Presence of Stromal Cell Conditioned Medium

Primary stromal cells derived from BPH or NAP were seeded into 6 cm dishes in complete medium. As soon as the stromal cells reached full confluency, the cells were cultured for an additional 2 days with fresh medium in the presence of 1 nM R1881 or vehicle prior to collection of conditioned medium (CM). CM was collected from 100% confluent plates by aspirating from the culture dish into a 15 mL falcon tube. The stromal CM was centrifuged at 180 x g for 5 min to remove cellular debris and the supernatant was collected and stored at 4°C. BPH‐1 or BHPrE1 (2500 cells) were mixed with 40 μL of Matrigel then added carefully at the center of each well of a 24‐well plate, and gels were allowed to solidify for 15 min at 37°C. After Matrigel solidification, 500 µL stromal CM plus 500 µL fresh complete medium were added to each well and cultured without disturbing for 2 days at 37°C. The media in each well was then replaced with a fresh mixture of 500 μL CM and 500 μL completed medium every 2 days; BPH‐1 or BHPrE1 cells were cultured in 3D culture for a total of 6 days. Epithelial spheroids (about 20 spheres/well) were imaged using an inverted phase–contrast microscope (Nikon TE2000‐U) equipped with Hamamatsu digital camera (Hamamatsu Inc) and NIS‐Elements Documentation v 4.6 software (Nikon Instruments Inc., Mellville, NY) with a 20x or 40x objective. On day 6, cells were fixed using 2% paraformaldehyde (PFA) at room temperature for 30 min. The diameter of the spheres was measured using ImageJ (Wayne Rasband, National Institutes of Health, USA).

### 2.8 Quantitative Real‐Time RT‐PCR (qPCR)

Validation of RNA‐seq results was performed with a separate experimental group of stromal cells treated as described for the RNA‐seq analysis experiment above. The RNA reverse transcription was carried out using the first‐strand cDNA synthesis kit (Promega, Madison, WI). Quantitative real‐time RT‐PCR (qPCR) was performed using SYBR green mix (Thermo Scientific, Rockford, IL). PCR amplification was carried out using Applied Biosystems StepOne™Plus™ Real‐Time PCR Systems (Applied Biosystems CA, USA). The expression of indicated genes was normalized with respect to the GAPDH mRNA level. Data were analyzed using a 2^‐ΔΔCt^ method. The primers for TNFRSF11B and IL32 are following: TNFRSF11B Forward: 5'‐ GTC TTT GGT CTC CTG CTA ACT C −3'; TNFRSF11B Reverse: 5' ‐ CCT CAC ACA GGG TAA CAT CTA TTC − 3'; IL‐32 Forward: 5' ‐ ATG TGC TTC CCG AAG GTC CTC TCT GA − 3'; and IL−32 Reverse: 5' ‐ TCA TTT TGA GGA TTG GGG TTC AGA GC − 3' Each experimental sample was assayed in triplicate.

### 2.9 Statistics and Calculations

Statistical analyses were performed with GraphPad Prism 7 (GraphPad Software, La Jolla, California, USA). Data were expressed as mean ± standard deviation. Differences between groups were analyzed by Multiple *t*‐tests‐one per row. Statistical significance was defined as *p* < 0.05.

## 3 Results

### 3.1 Androgen Receptor Protein in Primary BPH Stromal Cells Is Elevated in a Subset of Patients

To explore the androgen responsiveness of BPH stromal cells, we first examined androgen receptor (AR) protein level in primary BPH stromal cells, along with paired NAP stromal cells derived from the same patient. As shown in Figure 1, AR protein levels varied in primary BPH stromal cells derived from 7 different patients but in 4 paired cultures, AR levels appeared similar in NAP versus BPH stromal cell cultures. In contrast, AR level in NAP stromal cells from 3 of the 7 patients was very low or not detected. This finding suggests that AR expression in BPH stromal cells is elevated as compared to the paired NAP stromal cells in the same prostate in a subset of patients.

**Figure 1:**
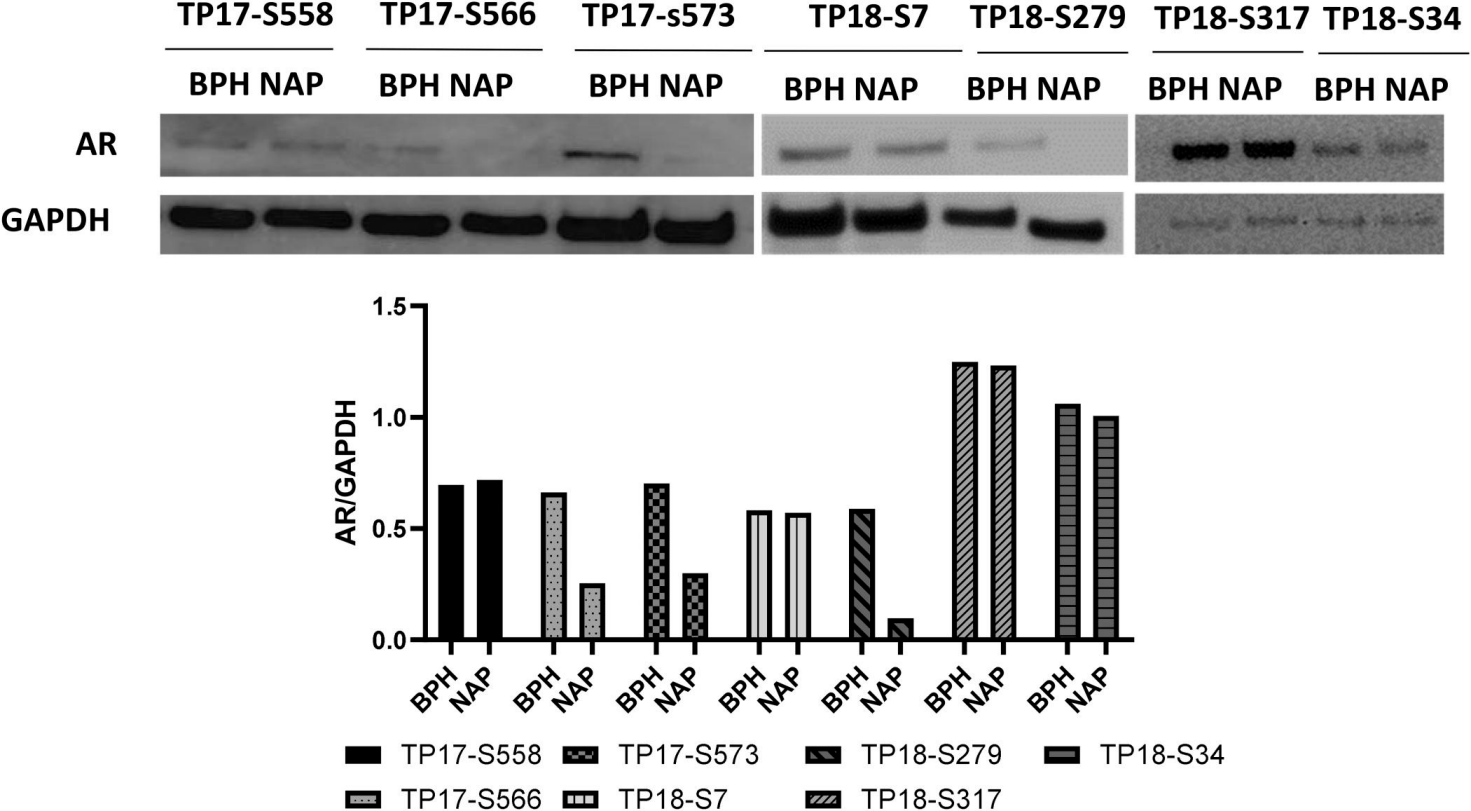
Upper panel shows Western blot analysis of stromal AR in paired primary stromal cells derived from BPH and normal adjacent prostate (NAP) of 7 different patients. Lower panel shows quantification of AR protein levels.

### 3.2 Androgen‐Induced Gene Expression in BPH Is Heightened Compared to NAP Primary Stromal Cells

To evaluate androgen signaling in BPH and NAP stromal cells, we performed RNA‐Seq and identified androgen‐response genes in paired primary BPH and NAP stromal cells from 3 different patients with BPH. Primary stromal cells were cultured in the presence of 1 nM R1881 with or without 10 μM MDV3100 (enzalutamide), a highly potent and specific AR antagonist. The expression of androgen‐response genes should be inhibited by enzalutamide. Principal component analysis (PCA) of the RNA‐seq data demonstrated clear clustering among different treatment groups. Interestingly, when stimulated with androgen, BPH stromal cells show greater sensitivity compared to NAP, as evidenced by the larger distance between the stimulated (BR = BPH with 1 nM R1881) and control groups (BM = BPH with 1 nM R1881 plus 10 μM MDV3100) for BPH samples on the PCA plot (Figure 2A). To corroborate this result, we further analyzed the differentially expressed genes (DEGs) in BPH and NAP samples from different patients with androgen stimulation. In all three patient samples, BPH showed more DEGs compared to NAP samples from the same patient (Figure 2B). Venn diagram shows the intersection of the up‐ (red) and down‐regulated (blue) genes in BPH samples from different patients after androgen stimulation (Figure 2C). The results show that seven genes (ALCAM, ANO3, GAS1, IRAG1, JPH2, NEDD9, RHOJ) are consistently up‐regulated across all samples, while three genes (CYP2S1, GAL, INSIG1) are consistently down‐regulated. Due to significant individual differences among patients, we define genes that are simultaneously up‐regulated in two or more patients as AR induced up‐regulated genes, and genes that are simultaneously down‐regulated in two or more patients as AR induced down‐regulated genes. In total, we identified 82 up‐regulated genes (Table S1) and 58 down‐regulated genes (Table S2). Notably, 17 of the 82 up‐regulated genes (21%) are known or predicted secreted proteins, representing a significant enrichment over the 7 of 58 (12%) in down‐regulated genes [19]. This suggests that AR can promote stromal cells to release more secreted proteins, thereby regulating neighboring cells in the microenvironment through paracrine signaling.

**Figure 2:**
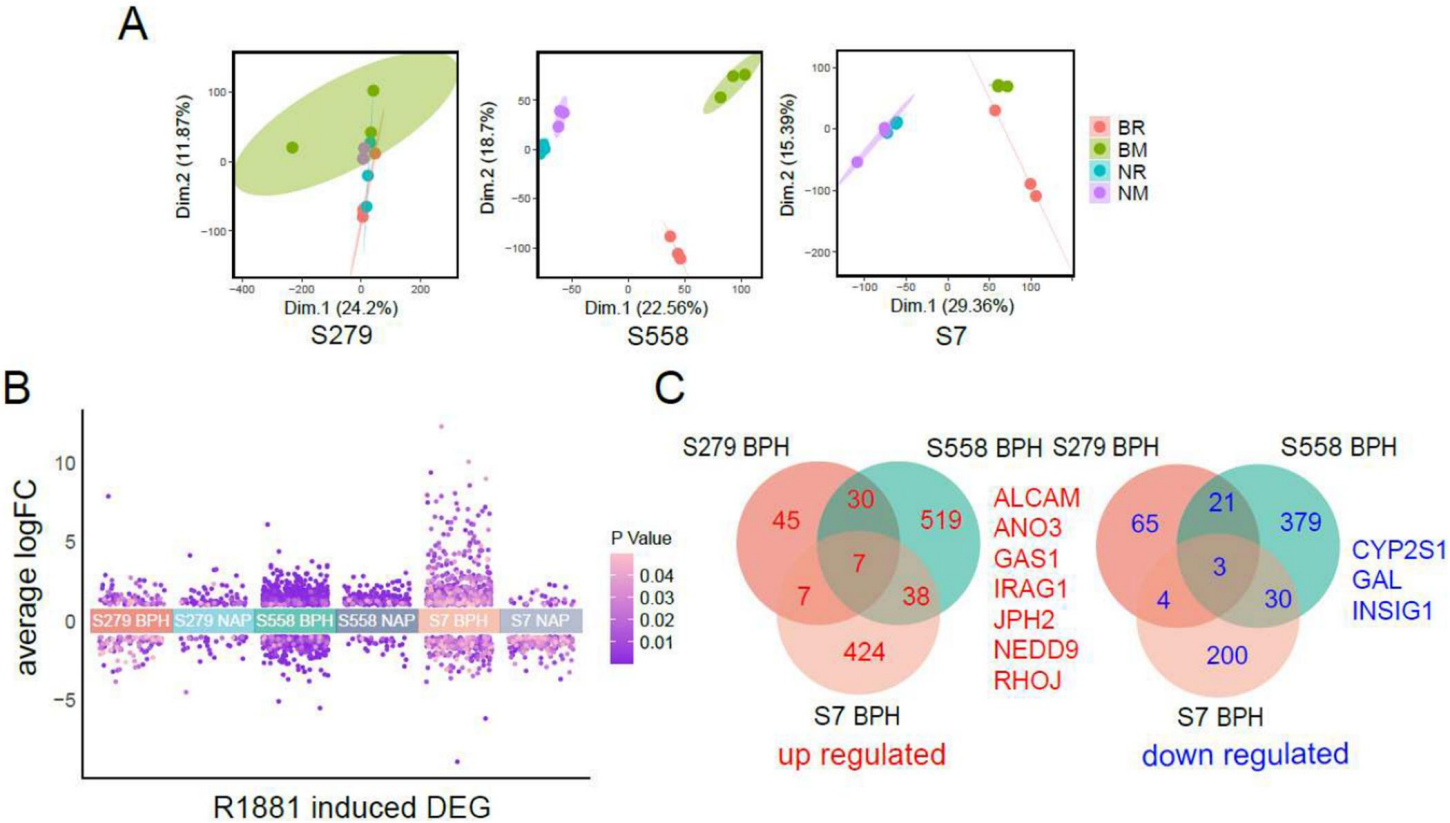
R1881‐induced transcriptomic changes in normal and BPH stromal cells. A. Principal component analysis (PCA) of TPM matrix corresponding to all NAP and BPH specimens. Each dot represents a sample, with dots of the same color indicating replicates of the same sample. B. Differential gene expression analysis reveals both up‐ and down‐regulated genes following androgen treatment across all specimens. The shade of the color represents the amount of *p*‐value. C. Venn diagram demonstrating common up‐regulated (red) and down‐regulated (blue) genes among different patient samples after androgen stimulation. BR = BPH cells treated with R1881; BM = BPH cells treated with both R1881 and MDV3100; NR = NAP cells treated with R1881; and NM = NAP cells treated with both R1881 and MDV3100. [Color figure can be viewed at wileyonlinelibrary.com]

GO analysis revealed that AR‐induced upregulated genes were mainly enriched in biological processes related to cell motility and migration, circulatory system and vascular development, and regulation of blood pressure and vasoconstriction, suggesting that androgen signaling may enhance stromal cell activity and promote paracrine‐mediated tissue remodeling. In contrast, the downregulated genes were significantly associated with ATP and nucleotide biosynthesis, mitotic regulation and cell cycle progression, and DNA repair and replication, indicating that androgen stimulation may influence stromal cell proliferation and metabolic activity (Figure 3A). To further explore the biological processes affected by androgen stimulation, we also performed KEGG pathway enrichment analysis using a broader set of DEGs, including genes differentially expressed in at least one patient, to capture more comprehensive androgen‐responsive pathways. The analysis revealed multiple pathways associated with cell proliferation, secretion, and intercellular communication, highlighting the diverse regulatory effects of AR signaling in stromal cells. Affected pathways included cell cycle, TGF‐beta signaling pathway, cytokine‐cytokine receptor interaction, Wnt signaling pathway, ECM‐receptor interaction, calcium signaling pathway, and vascular smooth muscle contraction, etc (Figure 3B). Many of these pathways are closely related to cell growth regulation and secretory activity. For instance, cell cycle and TGF‐beta signaling are central regulators of stromal cell proliferation, while cytokine‐cytokine receptor interaction and ECM‐receptor interaction are key mediators of paracrine communication between stromal and epithelial cells. Moreover, Wnt signaling and calcium signaling pathways are known to coordinate cell differentiation and contractile responses, both of which are critical for prostate tissue remodeling.

**Figure 3:**
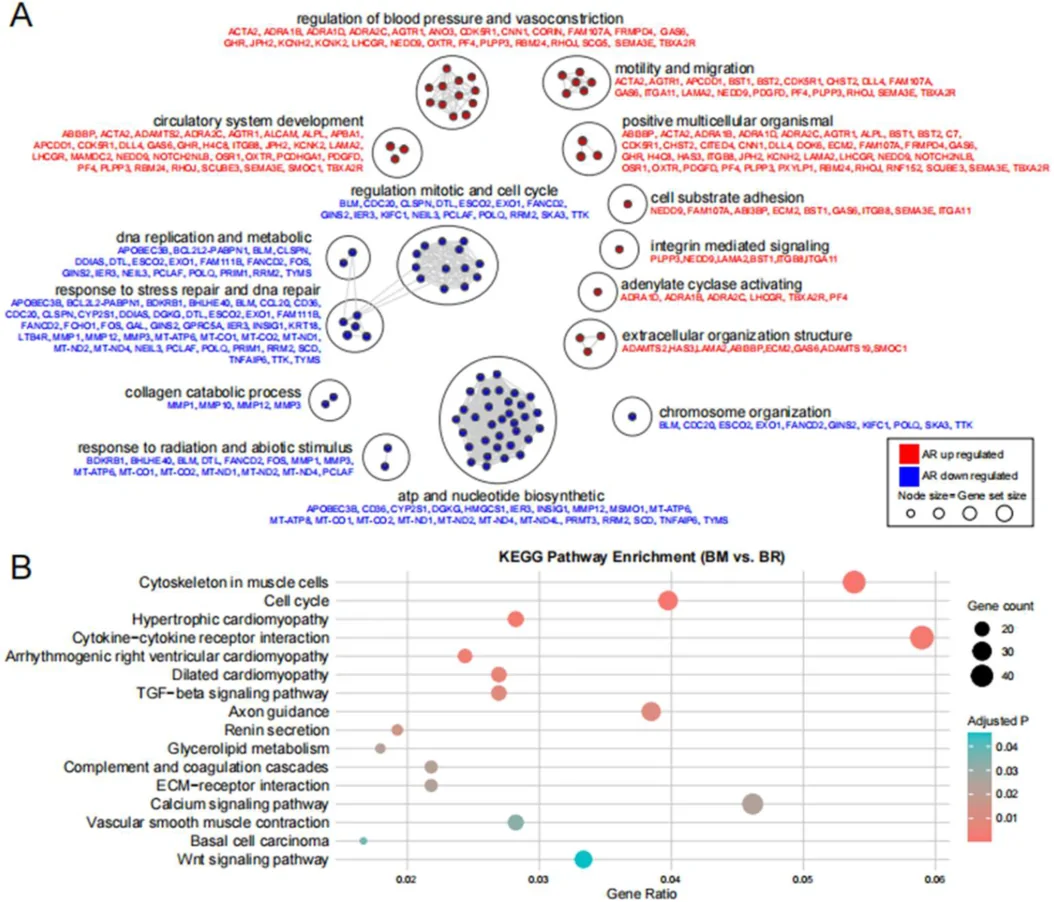
Functional enrichment of androgen‐regulated genes. A. GO biological process enrichment map for androgen up‐ (red) and down‐regulated (blue) genes. Node size reflects gene set size; edges indicate high similarity between gene sets, grouped into manually annotated subclusters. B. KEGG pathway enrichment. Gene Ratio (x‐axis) indicates the proportion of input genes in each pathway; dot size shows the number of enriched genes, and color represents adjusted p‐value (Benjamini–Hochberg correction). Pathways are ranked by significance. [Color figure can be viewed at wileyonlinelibrary.com]

Collectively, both GO and KEGG analyses consistently indicate that androgen signaling in BPH stromal cells exerts multifaceted regulatory effects. AR activation appears to enhance cellular motility, vascular and extracellular matrix remodeling, and paracrine signaling, while simultaneously modulating cell cycle and metabolic processes. These coordinated changes suggest a shift in stromal cells toward a more active, secretory, and signaling‐oriented state, which may enhance androgen‐dependent stromal–epithelial interactions and contribute to prostate tissue growth and the hyperplastic phenotype observed in BPH.

### 3.3 Comparative Transcriptomic Analysis of BPH and NAP Stromal Cells

We subsequently conducted a comparative transcriptomic analysis between paired primary BPH and NAP stromal cells from three patients. Volcano plots showed genes that are up‐regulated and down‐regulated in primary BPH stromal cells compared to primary NAP stromal cells both under AR blockade (BM vs NM; Figure 4A) and under androgen stimulation (BR vs NR; Figure S2) in different patients. Venn diagram showed the intersection of differential genes originating from different patients, with up‐regulation on the left and down‐regulation on the right (Figure 4B). Similarly, we selected DEGs that were consistently up‐ or down‐regulated in at least two patient samples for subsequent GO enrichment analysis, while the union of DEGs detected in at least one sample was used for KEGG pathway analysis. In total, we obtained 72 up‐regulated genes and 154 down‐regulated genes and selected the known or predicted secreted proteins among them. Among the 72 up‐regulated genes, we identified 18 secreted proteins that may play a crucial role in regulating epithelial cell proliferation in the microenvironment through paracrine signaling in BPH stromal cells (Tables [Link], [Link]). One of these, placental growth factor (PGF), has been reported to be highly expressed at the mRNA level in BPH stromal cells [20]. Further study of PGF and other secreted proteins may provide crucial insights into BPH pathogenesis. The GO analysis of signature genes in BPH revealed that most of these genes are associated with biological processes such as development, differentiation, and proliferation. This is consistent with the phenotype of uncontrolled abnormal growth observed in BPH (Figure 4C). Consistently, KEGG pathway enrichment analysis further highlighted signaling pathways related to cell proliferation, adhesion, and intercellular communication, including TGF‐beta signaling, PI3K‐Akt signaling, TNF signaling, Hippo signaling, and focal adhesion. In addition, pathways such as cytokine‐cytokine receptor interaction, ECM‐receptor interaction, and calcium signaling pathway were also enriched, suggesting enhanced stromal‐epithelial crosstalk and extracellular matrix remodeling. Together, these results indicate that dysregulation of these pathways may contribute to the excessive proliferative and structural remodeling activities characteristic of BPH (Figure 4D). To propose potential therapeutic options for BPH based on BPH stromal signature genes, we employed the Connectivity Map analysis. This approach leverages transcriptional expression data to explore connections between small molecules, genes, and disease [21, 22]. Figure 4E displays the top 10 nominated compounds according to the CMAP score. These compounds include antiviral agents and AR (androgen receptor) targeting drugs, which align with the characteristics of BPH and current clinical AR‐targeted treatments. Additionally, the list includes ERK inhibitors and BCL inhibitors, among others. This information provides valuable insights for subsequent experimental studies on BPH.

**Figure 4:**
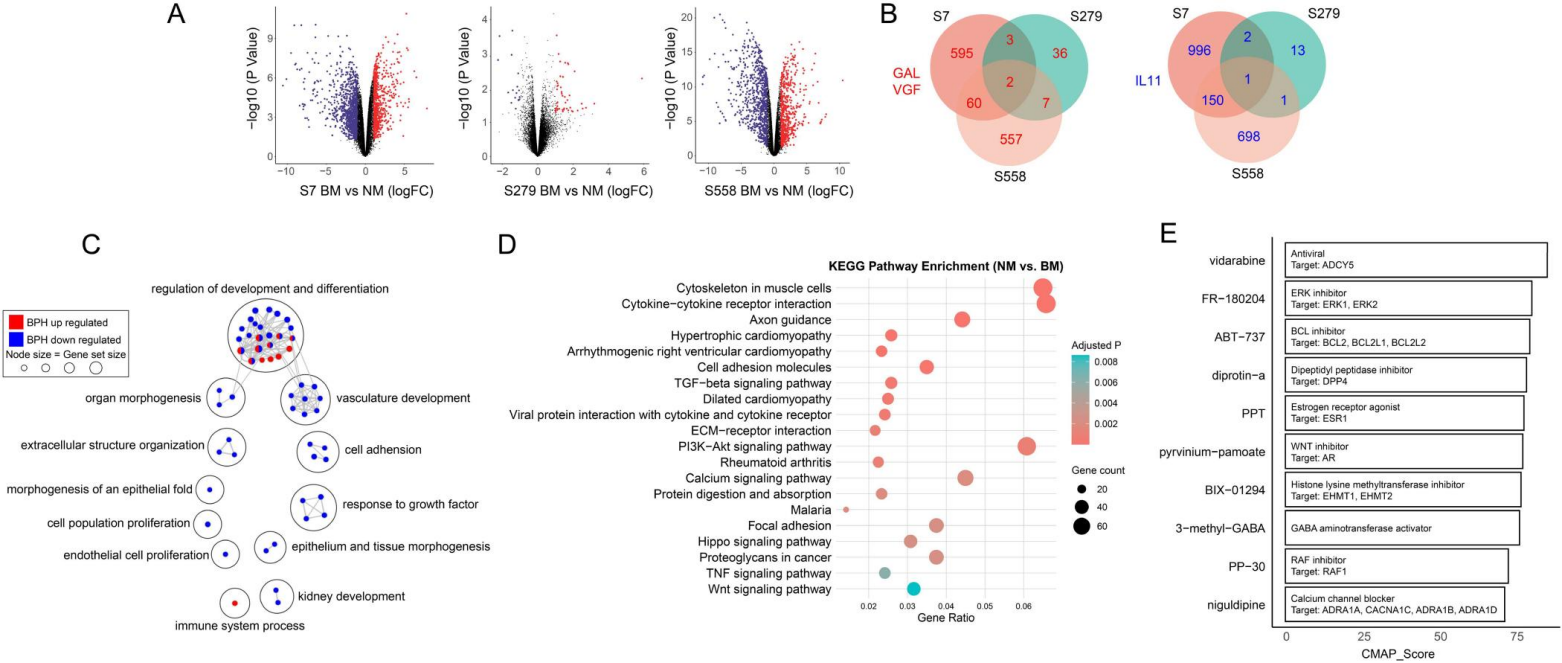
Transcriptome differences and small molecule drug prediction in BPH and NAP stromal cells. A. Volcano plots revealing up‐regulated genes (red) and down‐regulated genes (blue) in BPH stromal cells compared to NAP stromal cells across different patients. B. Venn diagram illustrating common up‐regulated (red) and down‐regulated (blue) genes among different patient BPH stromal cells compared to NAP stromal cells. C. Enrichment map of Gene Ontology (GO) terms within the ‘biological process’ category for BPH up‐regulated (red) and down‐regulated (blue) genes. Nodes represent gene sets, with node size corresponding to gene set size. Edges connect highly similar gene sets, which are grouped into subclusters and annotated manually. D. KEGG pathway enrichment analysis of BPH‐regulated genes. Gene Ratio (x‐axis) indicates the proportion of input genes in each pathway; dot size shows the number of enriched genes, and dot color represents adjusted p‐value (Benjamini–Hochberg correction). Pathways are ranked by significance. E. Top 10 nominated compounds from BPH identified via Connectivity Map. The x‐axis represents the CMAP score. [Color figure can be viewed at wileyonlinelibrary.com]

To support the results of RNA Seq, we assayed several cytokines, such as IL32 and TNFRSF11B, that were differentially expressed in paired primary stromal cells from a different patient (TP17‐S573) through RT‐PCR. RNA‐Seq showed IL32 and TNFRSF11B were suppressed in BPH stromal cells in the presence of MDV3100. Quantitative PCR analysis revealed that IL32 and TNFRSF11B were elevated in BPH stromal cells compared to NAP stromal cells, which was consistent with RNA‐Seq results (Figure 5). Quantitative PCR also showed androgen induction of these genes in primary BPH stromal cells, but not in NAP stromal cells (Figure 5).

**Figure 5:**
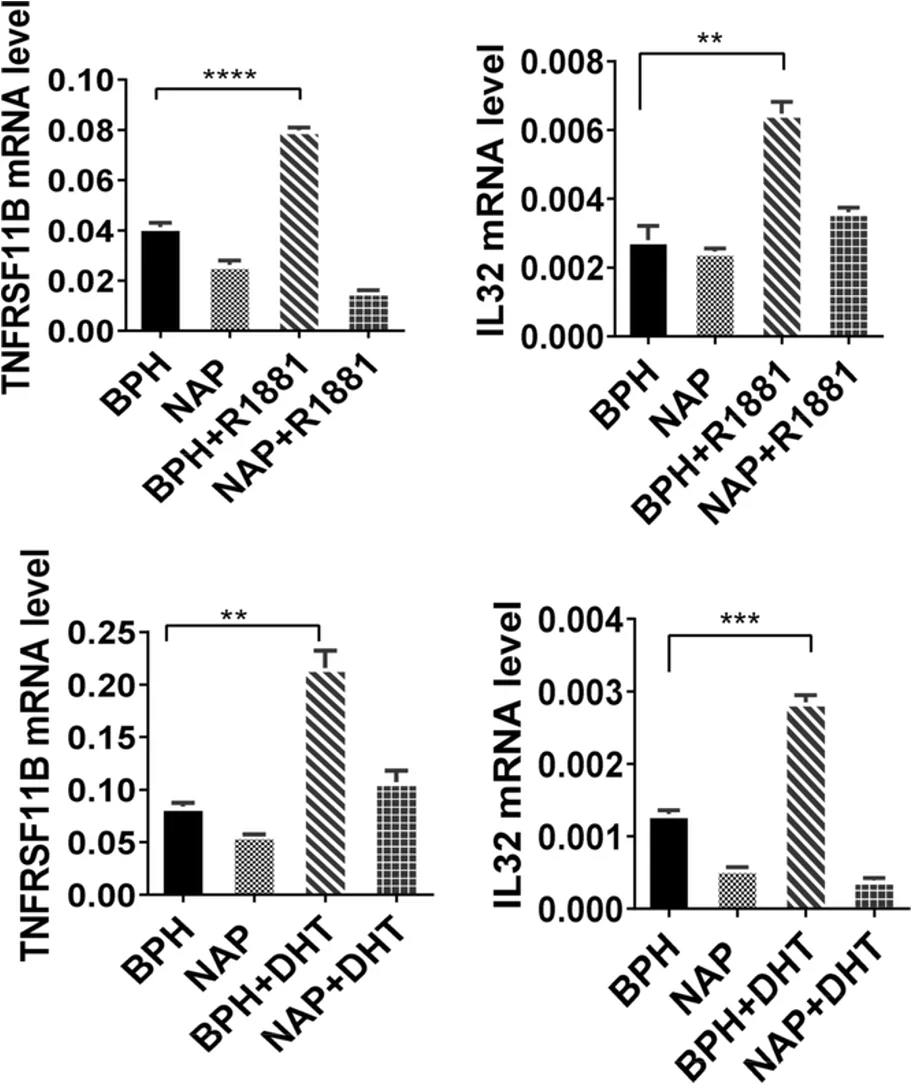
Quantitative PCR analysis of TNFRSF11B and IL32 mRNAs in BPH and NAP stromal cells derived from a different patient (TP17‐S573). The stromal cells were cultured in the absence or presence of 1 nM R1881 or 10 nM DHT for 48 h prior to RNA isolation. (*p < 0.05, **p < 0.01, ***p < 0.001).

### 3.4 Stimulation of Epithelial Proliferation by Androgen‐Treated BPH Stromal Conditioned Medium

To explore whether the differences in R1881‐induced secretory factor gene expression in RNASeq analysis of BPH vs NAP cells is associated with a functional consequence, we first tested the effect of synthetic androgen R1881 on prostatic epithelial cell lines BPH‐1 and BHPrE1, as well as on primary stromal cells, in monolayer culture. R1881 did not significantly stimulate the proliferation of any of the cultured cells in monolayer (data not shown). Next, we assessed the impact of R1881 on the growth of BPH‐1 and BHPrE1 cells in a 3D culture system. Cells were cultured in Matrigel for 6 days in the presence of conditioned medium (CM) from stromal cells derived from either BPH tissues or paired normal adjacent tissues (NAP), with or without 1 nM R1881. BHPrE1 cells cultured alone in Matrigel and treated with R1881 showed no enhanced growth (Figure 6A). Similarly, no significant difference in spheroidal size was observed when BHPrE1 cells were treated with CM from NAP stromal cells and R1881 (Figure 6B). However, when BHPrE1 cells were treated with CM from BPH stromal cells in the presence of R1881, the spheroid size significantly increased (Figure 6C). The same results were observed in BPH‐1 cell lines (Figure 6D–F). These findings were reproduced using another pair of stromal cells (Supplemental Fig. S1). This suggests that androgens, through their effects on BPH‐derived stromal cells, can induce the growth of epithelial cells.

**Figure 6:**
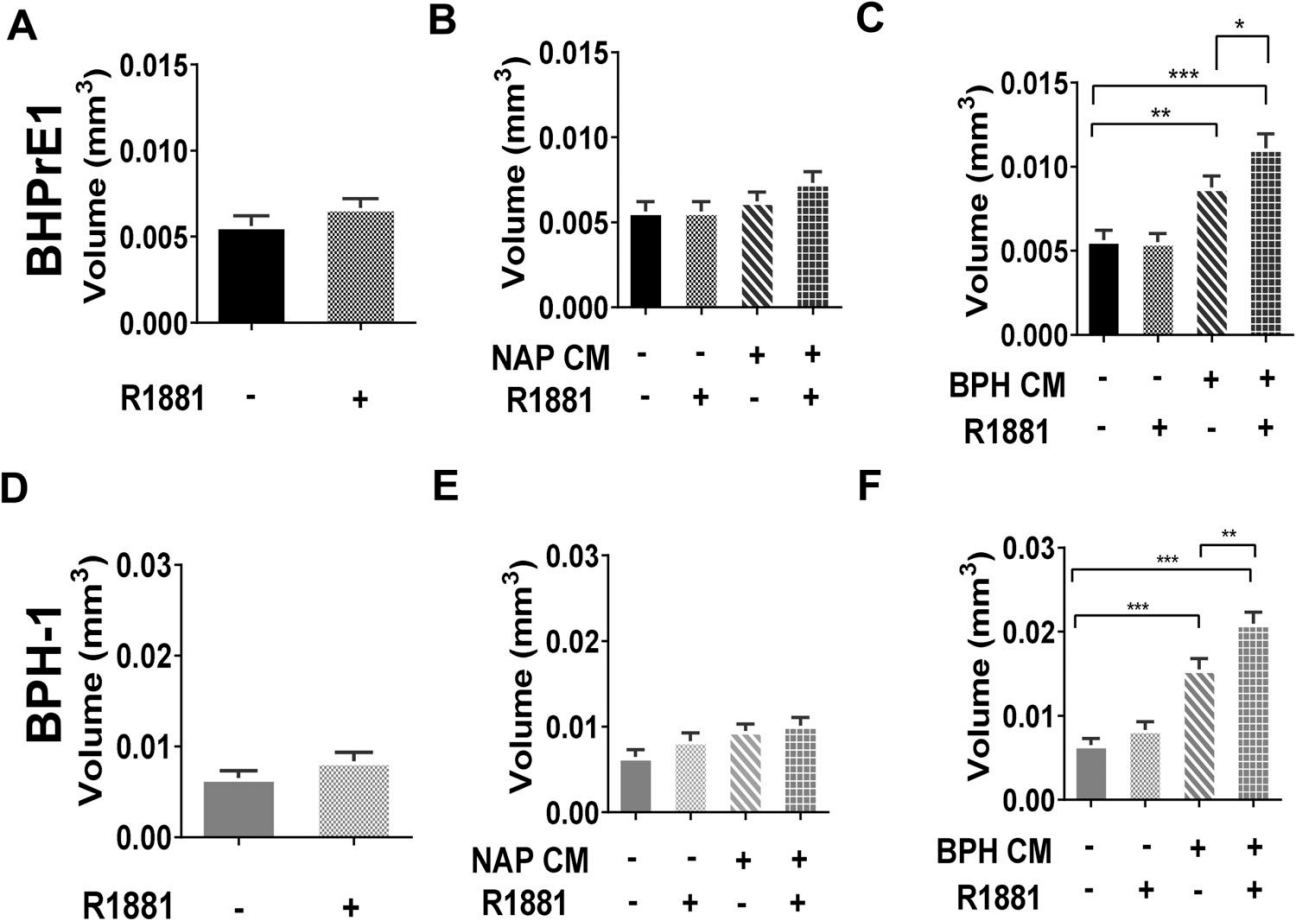
BPH stromal cells mediate androgen‐induced epithelial proliferation in 3D culture. A. Effect of R1881 on the spheroid growth of BHPrE1 cells in Matrigel. B. Spheroid growth of BHPrE1 cells in Matrigel in the presence or absence of conditioned medium (CM) from primary NAP stromal cells cultured with or without R1881. C. Spheroid growth of BHPrE1 cells in Matrigel in the presence or absence of CM from primary BPH stromal cells cultured with or without R1881. D. Effect of R1881 on the spheroid growth of BPH‐1 cells in Matrigel. E. Spheroid growth of BPH‐1 cells in Matrigel in the presence or absence of CM from primary NAP stromal cells cultured with or without R1881. F. Spheroid growth of BPH‐1 cells in Matrigel in the presence or absence of CM from primary BPH stromal cells cultured with or without R1881. The BPH and NAP stromal cells were derived from the same patient (TP17‐S558).

## 4 Discussion

Our studies showed that primary cultured BPH stromal cells exhibited heightened androgen responsiveness compared to their NAP counterpart. Also, primary BPH stromal cells can stimulate benign prostatic epithelial cell growth in 3D culture via androgen‐regulated paracrine signaling. In contrast, the paired NAP stromal cells did not stimulate benign prostatic epithelial cell growth in the parallel experiments. These findings suggest that BPH stromal cells play an essential role in stimulating androgen‐dependent prostatic epithelial cell proliferation in BPH development.

RNA‐Seq showed androgens can induce the expression of genes encoding secreted factors in BPH stromal cells. Thus, these secreted factors could mediate BPH stromal cells stimulation of prostatic epithelial proliferation. RNA‐Seq analysis also showed variabilities in transcriptomes in primary BPH stromal cells and NAP stromal cells from different BPH patients. Androgens appeared to induce different sets of androgen‐response genes in primary stromal cells from different patients. This raises the possibility that BPH stromal cells from different patients may use overlapping but different sets of secreted factors to stimulate prostatic epithelial cell proliferation. Multiple cytokines and growth factors have been reported to mediate stromal‐to‐epithelial interactions in the prostate [23]. It will be important to define the roles of multiple cytokines and growth factors in BPH pathogenesis.

BPH stromal cell stimulation of prostatic epithelial cell growth in the 3D culture (Figure 6) appears to be consistent with stromal induction of epithelial cell growth as part of the embryonic reawakening hypothesis [1, 11, 12]. BPH stromal cells have acquired the capability of stimulating prostatic epithelial cell proliferation via androgen‐regulated paracrine signaling. It will be interesting to test to what extent BPH stromal cells resemble embryonic prostatic stromal cells. In contrast, the paired primary NAP stromal cells were unable to stimulate prostatic epithelial cell proliferation. The inability of NAP stromal to stimulate prostatic epithelial growth is consistent with the fact that androgens do not stimulate proliferation in the normal prostate, although circulating androgens are required to maintain homeostasis of the intact normal prostate in adult man. Androgens only stimulate prostatic epithelial proliferation during regrowth of castrated normal prostate [24], presumably also via stromal AR [14, 15].

The mechanisms of increased sensitivity to androgens in BPH stromal cells are not clear. Higher AR expression in BPH stromal cells as compared to NAP stromal cells may represent one possible mechanism contributing to the heightened androgen‐responsive gene expression in BPH stromal cells. Notably, BPH vs NAP transcriptomic divergence did not simply reflect AR protein abundance. Patient S279, who showed the greatest AR elevation in BPH (Figure 1), had the fewest DEGs under both androgen‐stimulated (Fig. S2) and AR‐blocked (Figure 4A) conditions, while S7 and S558, with comparable BPH vs NAP AR levels, showed far greater divergence. This disconnect suggests that factors beyond AR abundance may contribute to the heightened androgen responsiveness of BPH stromal cells, warranting further investigation into the determinants of AR transcriptional output in this context. The paired primary BPH and NAP stromal cells provide an opportunity to explore the mechanisms leading to enhanced AR expression and function in BPH stromal cells.

BPH1 cells appeared to be more responsive to BPH CM than BHPrE1 cells in 3D culture. This difference may reflect the genetic and cellular differences between these two cell lines. BPH‐1 is an SV40 large T antigen‐immortalized, non‐tumorigenic human prostate epithelial cell line [25]. SV40 large T inactivates p53 and Rb tumor suppressors, which may enhance proliferative responsiveness to paracrine factors. BHPrE1 is a spontaneously immortalized benign prostate epithelial cell line with an intermediate cell phenotype (p63‐high/p53‐high/p21‐high/RB‐high) and CTNNB1 amplification [26]. Both cell lines carry genomic alterations associated with their respective immortalization mechanisms. Despite quantitative differences in spheroid volume, both lines showed the same qualitative response: androgen‐treated BPH stromal CM stimulated spheroid growth while NAP stromal CM did not (Figure 6).

Potential epithelial contamination in our study is very unlikely. Cultures were routinely monitored by microscopy throughout establishment and expansion. The cells consistently displayed the characteristic spindle‐shaped morphology of prostate stromal cells, and epithelial‐like colonies were not observed. In addition, primary prostate epithelial cells generally require more specialized culture conditions for survival and expansion and are less likely to persist under the stromal cell culture conditions used in this study. Therefore, significant epithelial contamination was considered unlikely. Importantly, E‐cadherin mRNA was not detected in our RNA‐seq analysis of the primary stromal cells, indicating that epithelial cells contamination is very unlikely.

In summary, this study suggested heightened androgen‐responsiveness of BPH stromal cells and stimulation of prostatic epithelial cell proliferation in 3D culture by androgen‐regulated paracrine signaling from primary stromal cells of BPH. These findings suggest that androgens can preferentially stimulate BPH epithelial cell growth indirectly via elevated androgen‐dependent paracrine signaling in BPH prostatic stromal cells.

## Author Contributions

Wei Chen, Fulu Dong, and Mohammad Reza Askarpour acquired data. Wei Chen, Fulu Dong, and Zhou Wang conducted data analysis and wrote the paper. Rajiv Dhir performed pathological analysis of BPH specimens. All authors contributed to the interpretation and reviewed and approved the final version.

## Conflicts of Interest

The authors declare no conflicts of interest.

## Supporting information


Supporting File 1

Supporting File 2

Supporting File 3

Supporting File 4

Supporting File 5

Supporting File 6

## Data Availability

The data that support the findings of this study are available on request from the corresponding author. The data are not publicly available due to privacy or ethical restrictions. Data supporting the results of this study are available from the corresponding author upon reasonable request.
