# Supplementary material for: Primary BPH Stromal Cell Cultures Exhibit Heightened Androgen‐Responsiveness
Source: Prostate. 2026 Aug 30;86(15):1508–17. doi: 10.1002/pros.70239 (PMC13656324; doi:10.1002/pros.70239)
Supplement: Supplementary file 1 [file PROS-86-1508-s001.pdf]

AR induced up regulated genes

| hgnc_symb | descriptior | S7_logFC | S7_PValue | S7_FDR | S279_logF | S279_PVal | S279_FDR | S558_logF | S558_PVal | S558_FDR | secreted p | patients_with_DEG |
|---|---|---|---|---|---|---|---|---|---|---|---|---|
| ABI3BP | ABI family r | 0.8599 | 1.179e-01 | 2.871e-01 | 1.605 | 1.664e-02 | 6.887e-01 | 1.5728 | 3.970e-05 | 2.073e-04 | yes | 2 |
| ACTA2 | actin alpha | -1.0239 | 1.407e-04 | 5.251e-02 | 1.0777 | 1.919e-03 | 4.939e-01 | 1.2046 | 2.285e-12 | 3.120e-10 | | 2 |
| ADAMTS19 | ADAM met | 7.9521 | 1.183e-02 | 1.039e-01 | 0.3716 | 2.940e-01 | 8.175e-01 | 1.8215 | 1.170e-10 | 6.746e-09 | yes | 2 |
| ADAMTS2 | ADAM met | 0.7325 | 1.367e-02 | 1.086e-01 | 1.4862 | 1.023e-05 | 1.291e-01 | 1.0716 | 2.343e-12 | 3.164e-10 | yes | 2 |
| ADRA1B | adrenocep | -0.7397 | 2.037e-02 | 1.248e-01 | 1.4108 | 8.349e-03 | 6.338e-01 | 2.8886 | 4.550e-11 | 3.226e-09 | | 2 |
| ADRA1D | adrenocep | 0.5695 | 1.388e-01 | 3.123e-01 | 1.9333 | 1.160e-03 | 3.881e-01 | 2.135 | 1.132e-08 | 2.563e-07 | | 2 |
| ADRA2C | adrenocep | 1.0651 | 1.851e-03 | 6.734e-02 | NA | NA | NA | 3.4314 | 1.090e-10 | 6.475e-09 | | 2 |
| AGTR1 | angiotensi | 4.6086 | 3.274e-02 | 1.516e-01 | -0.2373 | 3.820e-01 | 8.579e-01 | 1.2326 | 2.998e-03 | 8.267e-03 | | 2 |
| ALCAM | activated l | 1.9366 | 1.836e-02 | 1.210e-01 | 1.0279 | 6.843e-04 | 3.467e-01 | 1.5896 | 2.695e-13 | 6.651e-11 | yes | 3 |
| ALPL | alkaline ph | -0.206 | 6.592e-01 | 7.917e-01 | 2.304 | 1.099e-02 | 6.338e-01 | 1.8011 | 5.154e-06 | 3.951e-05 | | 2 |
| ANO3 | anoctamin | 4.1505 | 7.741e-03 | 9.228e-02 | 2.0457 | 4.788e-03 | 5.889e-01 | 2.5979 | 2.247e-04 | 9.124e-04 | | 3 |
| APBA1 | amyloid be | 2.6172 | 1.571e-02 | 1.142e-01 | 0.4104 | 4.077e-01 | 8.633e-01 | 1.6656 | 1.819e-08 | 3.784e-07 | | 2 |
| APCDD1 | APC down- | 1.0933 | 2.525e-02 | 1.362e-01 | 0.9732 | 1.357e-01 | 7.726e-01 | 4.3905 | 1.057e-14 | 5.899e-12 | | 2 |
| ATRNL1 | attractin lik | 3.1737 | 4.897e-02 | 1.810e-01 | NA | NA | NA | 2.2602 | 6.870e-08 | 1.117e-06 | | 2 |
| BST1 | bone marr | -1.1843 | 1.964e-05 | 3.341e-02 | 1.0223 | 2.328e-02 | 7.237e-01 | 1.588 | 4.267e-09 | 1.142e-07 | | 2 |
| BST2 | bone marr | 2.5363 | 3.581e-02 | 1.570e-01 | 0.3002 | 4.492e-01 | 8.719e-01 | 1.2314 | 6.873e-09 | 1.716e-07 | | 2 |
| C7 | compleme | 4.9571 | 1.306e-02 | 1.064e-01 | NA | NA | NA | 2.9975 | 4.093e-06 | 3.275e-05 | yes | 2 |
| CCDC85A | coiled-coil | NA | NA | NA | 1.0925 | 5.124e-03 | 5.985e-01 | 1.8572 | 5.339e-05 | 2.677e-04 | | 2 |
| CDK5R1 | cyclin depe | 1.1504 | 2.001e-02 | 1.241e-01 | 0.3143 | 5.096e-01 | 8.874e-01 | 2.0634 | 6.406e-07 | 7.020e-06 | | 2 |
| CHST2 | carbohydra | 3.5821 | 8.996e-03 | 9.631e-02 | 1.0452 | 7.916e-03 | 6.338e-01 | 0.9834 | 5.338e-07 | 6.061e-06 | | 2 |
| CITED4 | Cbp/p300 | 1.8939 | 1.150e-03 | 6.071e-02 | 0.7359 | 1.288e-01 | 7.726e-01 | 1.8784 | 1.691e-09 | 5.398e-08 | | 2 |
| CLGN | calmegin [S | 3.7541 | 9.585e-03 | 9.726e-02 | -0.1642 | 3.158e-01 | 8.261e-01 | 2.0845 | 3.714e-06 | 3.026e-05 | | 2 |
| CNN1 | calponin 1 | -0.6783 | 7.651e-04 | 5.669e-02 | 2.3874 | 1.549e-02 | 6.872e-01 | 3.3865 | 3.968e-10 | 1.714e-08 | | 2 |
| CORIN | corin, serir | 2.0876 | 5.092e-02 | 1.841e-01 | 1.6453 | 2.896e-04 | 3.467e-01 | 2.1662 | 5.505e-08 | 9.357e-07 | | 2 |
| COX7A1 | cytochrom | 1.8921 | 4.554e-03 | 7.929e-02 | 0.2432 | 1.856e-01 | 7.813e-01 | 1.5177 | 1.486e-03 | 4.552e-03 | | 2 |
| DLL4 | delta like c | 1.3239 | 2.777e-02 | 1.422e-01 | 1.2533 | 7.413e-04 | 3.596e-01 | -3.0863 | 5.370e-07 | 6.081e-06 | | 2 |
| DOK6 | docking pr | 5.7638 | 2.843e-02 | 1.436e-01 | 0.6018 | 3.885e-02 | 7.350e-01 | 1.2532 | 2.218e-03 | 6.392e-03 | | 2 |
| DUSP8P5 | dual specif | 1.2328 | 2.460e-02 | 1.343e-01 | 1.1664 | 1.864e-02 | 6.995e-01 | -0.3639 | 3.293e-01 | 4.248e-01 | | 2 |
| ECM2 | extracellul | -0.4984 | 1.594e-01 | 3.370e-01 | 1.7416 | 1.694e-02 | 6.887e-01 | 1.6024 | 3.043e-06 | 2.557e-05 | yes | 2 |
| FAM107A | family with | 0.892 | 5.274e-01 | 6.923e-01 | 2.1833 | 1.334e-02 | 6.757e-01 | 3.5206 | 3.245e-14 | 1.262e-11 | | 2 |
| FAM171A2 | family with | 1.004 | 4.163e-04 | 5.663e-02 | 0.514 | 2.099e-01 | 7.820e-01 | 1.7556 | 4.877e-08 | 8.480e-07 | | 2 |

| | | | | | | | | | | | | |
|---|---|---|---|---|---|---|---|---|---|---|---|---|
| FRMPD4 | FERM and | 2.0654 | 8.280e-03 | 9.366e-02 | 0.1133 | 6.810e-01 | 9.298e-01 | 1.0941 | 4.292e-06 | 3.410e-05 | | 2 |
| GAS1 | growth arre | 1.7855 | 7.810e-03 | 9.237e-02 | 1.5829 | 1.620e-03 | 4.643e-01 | 2.463 | 2.366e-14 | 1.006e-11 | | 3 |
| GAS6 | growth arre | 1.0411 | 2.998e-02 | 1.462e-01 | 0.8411 | 1.159e-01 | 7.704e-01 | 1.2297 | 2.759e-12 | 3.506e-10 | yes | 2 |
| GHR | growth hor | 1.3268 | 3.708e-02 | 1.594e-01 | 0.6195 | 4.378e-02 | 7.350e-01 | 1.6123 | 1.683e-11 | 1.612e-09 | yes | 2 |
| H3P6 | H3 histone | 3.9339 | 4.926e-02 | 1.817e-01 | 7.8813 | 5.154e-04 | 3.467e-01 | NA | NA | NA | | 2 |
| H4C8 | H4 cluster | 1.1124 | 2.792e-03 | 7.473e-02 | -0.036 | 8.882e-01 | 9.780e-01 | 1.3936 | 1.636e-06 | 1.530e-05 | | 2 |
| HAS3 | hyaluronar | 0.0395 | 9.421e-01 | 9.704e-01 | 2.2614 | 1.306e-04 | 3.467e-01 | 6.0952 | 3.297e-19 | 2.222e-15 | | 2 |
| HSD17B14 | hydroxyste | 7.0467 | 1.283e-02 | 1.061e-01 | -0.7514 | 1.059e-01 | 7.640e-01 | 1.0318 | 1.053e-03 | 3.402e-03 | | 2 |
| IMPA2 | inositol mc | 1.4123 | 3.286e-02 | 1.518e-01 | 0.0351 | 9.042e-01 | 9.821e-01 | 1.2909 | 2.573e-10 | 1.236e-08 | | 2 |
| INMT | indolethyla | 1.0972 | 6.821e-03 | 8.825e-02 | 1.6301 | 6.889e-03 | 6.338e-01 | 0.7739 | 9.319e-09 | 2.186e-07 | | 2 |
| IRAG1 | inositol 1,4 | 1.2244 | 1.104e-02 | 1.011e-01 | 1.2322 | 2.890e-04 | 3.467e-01 | 1.876 | 1.041e-13 | 3.243e-11 | | 3 |
| ITGA11 | integrin sul | 1.1251 | 5.550e-03 | 8.277e-02 | NA | NA | NA | 1.013 | 8.799e-03 | 2.051e-02 | | 2 |
| ITGB8 | integrin sul | 2.4751 | 3.453e-04 | 5.663e-02 | NA | NA | NA | 1.0649 | 2.599e-05 | 1.459e-04 | | 2 |
| JPH2 | junctophili | 1.3638 | 1.012e-02 | 9.845e-02 | 1.6199 | 3.358e-02 | 7.334e-01 | 1.7736 | 1.366e-04 | 5.938e-04 | | 3 |
| KCNH2 | potassium | -0.4117 | 6.600e-01 | 7.921e-01 | 1.373 | 3.886e-02 | 7.350e-01 | 1.673 | 4.772e-04 | 1.737e-03 | | 2 |
| KCNK2 | potassium | 2.1441 | 5.543e-03 | 8.277e-02 | 1.8385 | 2.154e-02 | 7.221e-01 | NA | NA | NA | | 2 |
| LAMA2 | laminin sul | 0.2224 | 1.174e-01 | 2.865e-01 | 1.0415 | 7.737e-03 | 6.338e-01 | 1.3245 | 1.444e-04 | 6.242e-04 | yes | 2 |
| LHCGR | luteinizing | 1.4187 | 2.109e-02 | 1.262e-01 | NA | NA | NA | 1.6662 | 4.763e-06 | 3.704e-05 | | 2 |
| MAMDC2 | MAM doma | 1.8615 | 1.506e-02 | 1.123e-01 | 0.5657 | 9.944e-02 | 7.616e-01 | 2.4526 | 1.450e-12 | 2.241e-10 | yes | 2 |
| MCTP2 | multiple C2 | 2.7589 | 1.171e-02 | 1.036e-01 | NA | NA | NA | 1.043 | 1.266e-07 | 1.869e-06 | | 2 |
| MRO | maestro [S | 0.3448 | 7.775e-01 | 8.692e-01 | 1.38 | 2.647e-02 | 7.237e-01 | 1.8396 | 1.386e-03 | 4.289e-03 | | 2 |
| NEDD9 | neural pre | 2.6976 | 6.851e-03 | 8.825e-02 | 1.7955 | 7.461e-03 | 6.338e-01 | 2.9208 | 5.095e-13 | 9.905e-11 | | 3 |
| NEURL1B | neuralized | -1.0771 | 4.701e-01 | 6.445e-01 | 1.0552 | 7.255e-03 | 6.338e-01 | 1.036 | 5.404e-05 | 2.707e-04 | | 2 |
| NOTCH2N | notch 2 N- | -1.0694 | 3.182e-02 | 1.500e-01 | 1.0589 | 1.161e-02 | 6.425e-01 | 1.386 | 7.931e-04 | 2.676e-03 | yes | 2 |
| OSR1 | odd-skippe | 0.8678 | 6.249e-04 | 5.669e-02 | 1.3574 | 2.921e-02 | 7.308e-01 | 1.5509 | 4.470e-12 | 5.228e-10 | | 2 |
| OXTR | oxytocin re | 0.5177 | 3.643e-01 | 5.520e-01 | 1.5425 | 3.158e-03 | 5.207e-01 | 1.3695 | 9.442e-06 | 6.400e-05 | | 2 |
| PCDHGA1 | protocadh | -0.0487 | 9.199e-01 | 9.578e-01 | 1.6589 | 2.481e-02 | 7.237e-01 | 1.2435 | 1.526e-03 | 4.662e-03 | | 2 |
| PDE1A | phosphodi | 8.5664 | 1.057e-02 | 9.993e-02 | 0.4945 | 6.023e-02 | 7.523e-01 | 2.5623 | 5.471e-05 | 2.735e-04 | | 2 |
| PDGFD | platelet de | -1.645 | 4.714e-02 | 1.781e-01 | 1.4956 | 2.627e-03 | 5.173e-01 | 1.9495 | 6.984e-12 | 7.716e-10 | yes | 2 |
| PDZRN3 | PDZ domai | -0.1146 | 7.982e-01 | 8.829e-01 | 1.1215 | 2.899e-03 | 5.173e-01 | 2.1249 | 1.361e-15 | 2.183e-12 | | 2 |
| PF4 | platelet fac | 3.0182 | 1.682e-04 | 5.251e-02 | NA | NA | NA | 1.3878 | 1.329e-02 | 2.890e-02 | yes | 2 |
| PLPP3 | phospholip | -0.2799 | 5.328e-01 | 6.965e-01 | 1.2618 | 6.309e-04 | 3.467e-01 | 1.414 | 1.087e-13 | 3.243e-11 | | 2 |
| PXYLP1 | 2-phospho | -0.4314 | 2.466e-01 | 4.366e-01 | 1.7073 | 2.791e-04 | 3.467e-01 | 2.047 | 4.481e-15 | 3.833e-12 | | 2 |

| | | | | | | | | | | | | |
|---|---|---|---|---|---|---|---|---|---|---|---|---|
| RBM24 | RNA bindir | 1.6273 | 2.063e-02 | 1.255e-01 | -0.0631 | 8.704e-01 | 9.740e-01 | 1.2231 | 1.188e-07 | 1.775e-06 | | 2 |
| RHOJ | ras homolc | 3.0615 | 4.359e-02 | 1.712e-01 | 1.3458 | 1.929e-02 | 6.995e-01 | 1.0458 | 1.780e-02 | 3.685e-02 | | 3 |
| RNF150 | ring finger ( | 1.4984 | 2.148e-02 | 1.269e-01 | 0.1568 | 4.206e-01 | 8.651e-01 | 2.4968 | 2.265e-06 | 1.993e-05 | | 2 |
| RNF152 | ring finger ( | 1.7912 | 2.793e-03 | 7.473e-02 | 0.5182 | 2.820e-01 | 8.085e-01 | 1.3217 | 2.372e-06 | 2.072e-05 | | 2 |
| SCG5 | secretogra | 2.1268 | 2.127e-02 | 1.264e-01 | 0.5518 | 2.528e-01 | 7.948e-01 | 1.3563 | 8.266e-04 | 2.766e-03 | yes | 2 |
| SCUBE3 | signal pept | -0.2361 | 1.265e-01 | 2.967e-01 | 1.2582 | 6.041e-03 | 6.164e-01 | 1.6519 | 2.683e-12 | 3.443e-10 | yes | 2 |
| SEMA3E | semaphori | 4.0987 | 2.183e-03 | 6.923e-02 | 0.6752 | 1.242e-02 | 6.584e-01 | 1.709 | 1.251e-03 | 3.940e-03 | yes | 2 |
| SLC27A6 | solute carr | 3.7452 | 3.794e-02 | 1.610e-01 | -0.0485 | 8.228e-01 | 9.645e-01 | 1.374 | 6.377e-09 | 1.627e-07 | | 2 |
| SLC7A14 | solute carr | -0.477 | 5.806e-01 | 7.347e-01 | 1.6474 | 1.101e-02 | 6.338e-01 | 1.9309 | 8.162e-06 | 5.705e-05 | | 2 |
| SMOC1 | SPARC rela | 1.7953 | 2.209e-03 | 6.939e-02 | -0.1391 | 5.192e-01 | 8.890e-01 | 1.1291 | 5.181e-06 | 3.967e-05 | yes | 2 |
| SPRN | shadow of | 1.6151 | 3.530e-03 | 7.690e-02 | 0.3538 | 6.678e-02 | 7.616e-01 | 1.0479 | 4.949e-03 | 1.265e-02 | | 2 |
| SYT16 | synaptotag | 0.9407 | 1.197e-01 | 2.890e-01 | 1.2535 | 2.273e-02 | 7.221e-01 | 2.9302 | 8.892e-07 | 9.299e-06 | | 2 |
| TAGLN | transgelin ( | -0.6998 | 1.435e-03 | 6.258e-02 | 1.3261 | 2.697e-03 | 5.173e-01 | 1.9368 | 1.899e-15 | 2.481e-12 | | 2 |
| TBXA2R | thromboxa | 2.8482 | 9.369e-03 | 9.670e-02 | 0.4643 | 3.717e-01 | 8.525e-01 | 1.4105 | 1.377e-05 | 8.640e-05 | | 2 |
| TCEAL7 | transcriptio | 2.0072 | 1.490e-02 | 1.120e-01 | -0.159 | 4.480e-01 | 8.716e-01 | 1.3258 | 1.999e-05 | 1.181e-04 | | 2 |
| TMEM200A | transmeml | 0.2983 | 2.825e-01 | 4.707e-01 | 1.1372 | 6.498e-03 | 6.256e-01 | 1.5537 | 5.498e-08 | 9.357e-07 | | 2 |
| TRMT9B | tRNA meth | 2.7264 | 1.855e-02 | 1.213e-01 | 1.2271 | 1.364e-02 | 6.766e-01 | 0.5233 | 2.463e-01 | 3.361e-01 | | 2 |
| TSPAN9 | tetraspanir | 1.1779 | 6.526e-04 | 5.669e-02 | 0.3997 | 4.242e-02 | 7.350e-01 | 1.2933 | 1.810e-13 | 5.050e-11 | | 2 |
