# Supplementary material for: Primary BPH Stromal Cell Cultures Exhibit Heightened Androgen‐Responsiveness
Source: Prostate. 2026 Aug 30;86(15):1508–17. doi: 10.1002/pros.70239 (PMC13656324; doi:10.1002/pros.70239)
Supplement: Supplementary file 2 [file PROS-86-1508-s002.pdf]

AR induced down regulated genes

| hgnc_symb | descriptior | S7_logFC | S7_PValue | S7_FDR | S279_logF | S279_PVal | S279_FDR | S558_logF | S558_PVal | S558_FDR | secreted p | patients_with_DEG |
|---|---|---|---|---|---|---|---|---|---|---|---|---|
| APOBEC3E | apolipopro | -1.6502 | 4.725e-02 | 1.784e-01 | 0.6997 | 2.122e-01 | 7.820e-01 | -1.2636 | 1.231e-06 | 1.220e-05 | | 2 |
| BCL2L2-PA | BCL2L2-PA | 0.4923 | 5.509e-01 | 7.115e-01 | -1.1854 | 1.001e-02 | 6.338e-01 | -1.1169 | 1.224e-05 | 7.883e-05 | | 2 |
| BDKRB1 | bradykinin | -1.8059 | 4.744e-03 | 8.018e-02 | -0.2369 | 6.415e-01 | 9.217e-01 | -1.5636 | 3.458e-10 | 1.541e-08 | | 2 |
| BHLHE40 | basic helix | -0.8668 | 4.048e-04 | 5.663e-02 | -1.454 | 3.208e-02 | 7.334e-01 | -1.0858 | 3.785e-10 | 1.650e-08 | | 2 |
| BLM | BLM RecQ | -1.4421 | 2.372e-02 | 1.322e-01 | 0.8472 | 6.902e-02 | 7.616e-01 | -1.6186 | 5.337e-04 | 1.915e-03 | | 2 |
| CCL20 | C-C motif c | 1.9423 | 7.858e-02 | 2.316e-01 | -1.8347 | 7.636e-03 | 6.338e-01 | -3.2179 | 1.292e-05 | 8.226e-05 | yes | 2 |
| CD36 | CD36 mole | NA | NA | NA | -1.0301 | 1.153e-02 | 6.425e-01 | -1.2065 | 1.420e-02 | 3.058e-02 | | 2 |
| CDC20 | cell divisio | -2.0806 | 4.159e-02 | 1.674e-01 | 0.8988 | 1.893e-01 | 7.813e-01 | -1.016 | 5.495e-06 | 4.143e-05 | | 2 |
| CDCA7 | cell divisio | -2.1933 | 2.277e-02 | 1.308e-01 | 0.4689 | 8.676e-02 | 7.616e-01 | -1.3172 | 3.270e-08 | 6.029e-07 | | 2 |
| CLSPN | claspin [Sc | -1.2923 | 2.373e-02 | 1.322e-01 | 0.5372 | 3.607e-01 | 8.453e-01 | -1.5454 | 1.485e-08 | 3.224e-07 | | 2 |
| CYP2S1 | cytochrom | -1.5385 | 9.783e-04 | 5.994e-02 | -1.2494 | 4.706e-02 | 7.352e-01 | -1.636 | 1.847e-11 | 1.635e-09 | | 3 |
| DDIAS | DNA dama | -1.2795 | 3.088e-02 | 1.484e-01 | 0.8937 | 1.222e-01 | 7.706e-01 | -1.3552 | 2.068e-04 | 8.501e-04 | | 2 |
| DGKG | diacylglyce | -1.059 | 3.915e-02 | 1.627e-01 | 0.202 | 7.268e-01 | 9.405e-01 | -1.8108 | 4.933e-04 | 1.789e-03 | | 2 |
| DTL | denticleles | -1.3451 | 1.410e-03 | 6.247e-02 | 0.8796 | 2.868e-02 | 7.308e-01 | -1.6989 | 6.045e-07 | 6.692e-06 | | 2 |
| ESCO2 | establishm | -1.9608 | 2.537e-02 | 1.366e-01 | 1.059 | 2.642e-02 | 7.237e-01 | -1.3069 | 1.084e-04 | 4.869e-04 | | 2 |
| EXO1 | exonuclea: | -1.5453 | 1.071e-02 | 1.003e-01 | 0.7946 | 8.873e-02 | 7.616e-01 | -1.434 | 3.042e-06 | 2.557e-05 | | 2 |
| FAM111B | FAM111 try | -1.2062 | 1.449e-02 | 1.109e-01 | 1.1626 | 1.955e-01 | 7.813e-01 | -1.3009 | 2.243e-05 | 1.294e-04 | | 2 |
| FAM216A | family with | -1.0122 | 6.624e-03 | 8.722e-02 | 0.0871 | 7.836e-01 | 9.571e-01 | -1.0623 | 4.476e-07 | 5.226e-06 | | 2 |
| FANCD2 | FA comple | -1.1625 | 1.221e-03 | 6.142e-02 | 0.8895 | 1.026e-01 | 7.616e-01 | -1.26 | 5.127e-06 | 3.935e-05 | | 2 |
| FCHO1 | FCH and m | NA | NA | NA | -1.9198 | 2.716e-03 | 5.173e-01 | -1.069 | 1.257e-02 | 2.758e-02 | | 2 |
| FOS | Fos proto-c | NA | NA | NA | -1.8714 | 1.582e-02 | 6.887e-01 | -5.0912 | 1.294e-12 | 2.076e-10 | | 2 |
| GAL | galanin and | -2.3546 | 3.603e-02 | 1.575e-01 | -1.9137 | 2.546e-04 | 3.467e-01 | -2.84 | 2.194e-09 | 6.788e-08 | yes | 3 |
| GINS2 | GINS comp | -1.1628 | 3.493e-02 | 1.557e-01 | 0.5617 | 3.302e-01 | 8.307e-01 | -1.4179 | 1.552e-06 | 1.469e-05 | | 2 |
| GPRC5A | G protein-c | -1.1746 | 2.117e-05 | 3.341e-02 | -0.2852 | 4.128e-01 | 8.634e-01 | -1.5567 | 1.427e-14 | 7.326e-12 | | 2 |
| HMGCS1 | 3-hydroxy- | -0.7815 | 5.496e-03 | 8.273e-02 | -2.9406 | 2.843e-02 | 7.308e-01 | -1.2113 | 2.823e-10 | 1.332e-08 | | 2 |
| IER3 | immediate | 0.5392 | 1.181e-02 | 1.039e-01 | -1.6271 | 1.840e-02 | 6.995e-01 | -1.9867 | 6.693e-13 | 1.227e-10 | | 2 |
| INSIG1 | insulin indu | -1.614 | 5.776e-07 | 3.645e-03 | -3.8434 | 2.272e-02 | 7.221e-01 | -1.2357 | 2.301e-10 | 1.127e-08 | | 3 |
| KIFC1 | kinesin fan | -1.5214 | 5.576e-03 | 8.277e-02 | 0.8093 | 3.964e-02 | 7.350e-01 | -1.0375 | 5.337e-04 | 1.915e-03 | | 2 |
| KRT18 | keratin 18 \| | -1.3667 | 1.831e-02 | 1.210e-01 | 0.3559 | 2.025e-01 | 7.813e-01 | -1.1356 | 4.647e-12 | 5.372e-10 | | 2 |
| LTB4R | leukotriene | 1.0637 | 1.073e-02 | 1.003e-01 | -1.4472 | 4.329e-02 | 7.350e-01 | -1.4115 | 4.595e-05 | 2.343e-04 | | 2 |
| MMP1 | matrix met | 0.4874 | 4.310e-02 | 1.700e-01 | -2.8919 | 1.644e-02 | 6.887e-01 | -1.8573 | 1.037e-08 | 2.388e-07 | yes | 2 |

| | | | | | | | | | | | | |
|---|---|---|---|---|---|---|---|---|---|---|---|---|
| MMP10 | matrix met | -1.4847 | 2.082e-03 | 6.862e-02 | -2.7618 | 1.368e-03 | 4.108e-01 | NA | NA | NA | yes | 2 |
| MMP12 | matrix met | -1.7406 | 4.605e-02 | 1.759e-01 | -1.826 | 9.197e-03 | 6.338e-01 | NA | NA | NA | yes | 2 |
| MMP3 | matrix met | -0.5249 | 1.860e-01 | 3.685e-01 | -1.1097 | 2.222e-02 | 7.221e-01 | -1.2784 | 4.073e-06 | 3.268e-05 | yes | 2 |
| MSMO1 | methylster | -1.2198 | 4.022e-04 | 5.663e-02 | -2.1055 | 4.241e-02 | 7.350e-01 | -0.6532 | 1.490e-06 | 1.427e-05 | | 2 |
| MT-ATP6 | mitochond | 1.6983 | 4.165e-03 | 7.831e-02 | -1.1425 | 3.279e-02 | 7.334e-01 | -1.8776 | 1.021e-02 | 2.322e-02 | | 2 |
| MT-ATP8 | mitochond | 1.4152 | 5.495e-03 | 8.273e-02 | -1.0919 | 4.655e-02 | 7.350e-01 | -2.1532 | 1.281e-10 | 7.241e-09 | | 2 |
| MT-CO1 | mitochond | 1.1704 | 1.160e-02 | 1.031e-01 | -1.2359 | 6.469e-04 | 3.467e-01 | -1.4229 | 1.817e-02 | 3.748e-02 | | 2 |
| MT-CO2 | mitochond | 1.2747 | 2.314e-03 | 7.068e-02 | -1.2973 | 9.418e-03 | 6.338e-01 | -1.7716 | 2.713e-02 | 5.260e-02 | | 2 |
| MT-ND1 | mitochond | 1.6604 | 2.108e-03 | 6.875e-02 | -1.4597 | 3.973e-03 | 5.706e-01 | -1.4986 | 3.363e-02 | 6.304e-02 | | 2 |
| MT-ND2 | mitochond | 1.7703 | 2.902e-03 | 7.506e-02 | -1.2097 | 1.056e-02 | 6.338e-01 | -2.1109 | 9.733e-03 | 2.230e-02 | | 2 |
| MT-ND4 | mitochond | 1.6492 | 4.611e-03 | 7.977e-02 | -1.4343 | 1.255e-03 | 3.957e-01 | -1.5745 | 3.987e-02 | 7.269e-02 | | 2 |
| MT-ND4L | mitochond | 1.8123 | 3.600e-03 | 7.690e-02 | -1.245 | 8.040e-03 | 6.338e-01 | -1.79 | 5.639e-03 | 1.408e-02 | | 2 |
| MTND2P28 | MT-ND2 ps | 1.8654 | 4.892e-03 | 8.040e-02 | -1.2424 | 3.332e-02 | 7.334e-01 | -1.9101 | 3.599e-04 | 1.362e-03 | | 2 |
| NEIL3 | nei like DN | -1.6918 | 8.478e-03 | 9.412e-02 | 0.5865 | 5.284e-01 | 8.904e-01 | -1.1021 | 1.542e-03 | 4.707e-03 | | 2 |
| PCLAF | PCNA clam | -1.3779 | 1.553e-02 | 1.135e-01 | -0.2925 | 5.505e-01 | 8.955e-01 | -1.074 | 7.721e-06 | 5.468e-05 | | 2 |
| POLQ | DNA polym | -1.1059 | 1.358e-02 | 1.083e-01 | 0.7154 | 1.218e-01 | 7.706e-01 | -1.0951 | 3.520e-04 | 1.337e-03 | | 2 |
| PRIM1 | DNA prima | -1.459 | 1.030e-02 | 9.895e-02 | 0.4366 | 2.617e-01 | 7.986e-01 | -1.2141 | 1.095e-04 | 4.906e-04 | | 2 |
| PRMT3 | protein arg | -1.0446 | 1.005e-02 | 9.843e-02 | -0.1105 | 6.631e-01 | 9.266e-01 | -1.0305 | 1.222e-06 | 1.213e-05 | | 2 |
| PTMA | prothymos | -1.2906 | 1.325e-02 | 1.068e-01 | 0.085 | 5.565e-01 | 8.977e-01 | -1.4492 | 1.437e-12 | 2.241e-10 | | 2 |
| RPL22L1 | ribosomal | -1.0448 | 7.628e-04 | 5.669e-02 | 0.0078 | 9.757e-01 | 9.936e-01 | -1.1524 | 7.743e-09 | 1.882e-07 | | 2 |
| RRM2 | ribonucleo | -2.0412 | 1.050e-02 | 9.957e-02 | 0.8768 | 1.544e-01 | 7.808e-01 | -1.145 | 2.700e-08 | 5.187e-07 | | 2 |
| SCD | stearoyl-C | -1.3824 | 1.148e-02 | 1.026e-01 | -1.0615 | 2.158e-02 | 7.221e-01 | -0.8451 | 1.790e-09 | 5.672e-08 | | 2 |
| SKA3 | spindle and | -1.663 | 2.839e-03 | 7.496e-02 | 0.3107 | 6.522e-01 | 9.231e-01 | -1.1882 | 4.197e-05 | 2.177e-04 | | 2 |
| TNFAIP6 | TNF alpha i | -0.8776 | 2.906e-02 | 1.448e-01 | -1.6311 | 2.238e-03 | 5.173e-01 | -1.1652 | 8.846e-06 | 6.080e-05 | yes | 2 |
| TRBC2 | T cell recep | -1.2974 | 1.117e-02 | 1.013e-01 | NA | NA | NA | -2.4579 | 1.163e-04 | 5.160e-04 | | 2 |
| TTK | TTK proteir | -1.6489 | 4.440e-02 | 1.728e-01 | 0.5895 | 1.241e-01 | 7.726e-01 | -1.0347 | 2.217e-04 | 9.022e-04 | | 2 |
| TYMS | thymidylat | -1.483 | 2.432e-02 | 1.337e-01 | 0.2732 | 4.278e-01 | 8.671e-01 | -1.0662 | 3.624e-09 | 1.007e-07 | | 2 |
