# Supplementary material for: Primary BPH Stromal Cell Cultures Exhibit Heightened Androgen‐Responsiveness
Source: Prostate. 2026 Aug 30;86(15):1508–17. doi: 10.1002/pros.70239 (PMC13656324; doi:10.1002/pros.70239)
Supplement: Supplementary file 3 [file PROS-86-1508-s003.pdf]

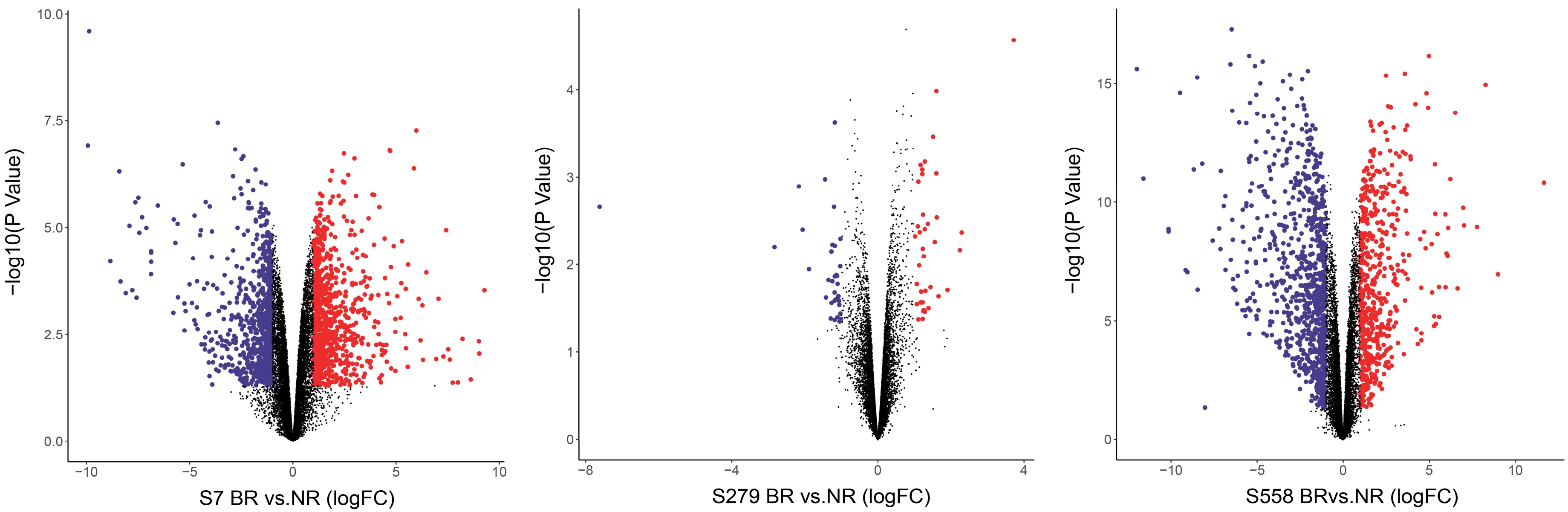

Figure S2. Transcriptomic differences between BPH and NAP stromal cells under androgen stimulation. Volcano plots showing differentially expressed genes in BR vs NR comparisons across three patients. Red dots: upregulated genes (log2fold change ≥ 1, $P < 0.05$); blue dots: downregulated genes (log2fold change ≤ −1, $P < 0.05$); black dots: not significant.
