# Supplementary material for: Primary BPH Stromal Cell Cultures Exhibit Heightened Androgen‐Responsiveness
Source: Prostate. 2026 Aug 30;86(15):1508–17. doi: 10.1002/pros.70239 (PMC13656324; doi:10.1002/pros.70239)
Supplement: Supplementary file 4 [file PROS-86-1508-s004.pdf]

Down regulated genes in BPH

hgnc_symb	descriptior	S7_logFC	S7_PValue	S7_FDR	S279_logF	S279_PVal	S279_FDR	S558_logF	S558_PVal	S558_FDR	secreted p	patients_with_DEG
ABCB1	ATP bindin	-6.2099	1.641e-06	8.692e-05	0.23	2.304e-01	8.962e-01	-4.3695	8.291e-14	8.714e-12		2
ADAMTS19	ADAM met	-7.8162	1.156e-06	6.881e-05	0.2878	3.645e-01	8.984e-01	-1.4213	5.721e-11	1.882e-09	yes	2
AGTR1	angiotensi	-7.0282	5.030e-05	6.319e-04	0.3384	2.112e-01	8.962e-01	-2.1513	2.122e-06	1.708e-05		2
AMPH	amphiphys	-3.1466	9.022e-04	4.287e-03	NA	NA	NA	-1.4086	2.894e-03	8.422e-03		2
ANKRD29	ankyrin rep	-1.0247	3.157e-02	6.263e-02	0.2362	3.945e-02	8.962e-01	-1.1224	2.223e-06	1.779e-05		2
ATP10A	ATPase ph	-3.2593	2.200e-04	1.614e-03	-0.1411	2.782e-01	8.964e-01	-6.9445	2.151e-14	2.927e-12		2
B4GALNT4	beta-1,4-N	-1.6407	1.001e-02	2.552e-02	0.1352	8.068e-01	9.671e-01	-1.6095	7.939e-03	1.985e-02		2
BEX4	brain expre	-3.0552	8.401e-06	2.101e-04	0.2348	2.489e-01	8.962e-01	-3.9984	1.291e-13	1.218e-11		2
C7	compleme	-6.045	1.927e-06	9.462e-05	NA	NA	NA	-2.8238	5.874e-06	4.080e-05	yes	2
CA9	carbonic a	-2.8848	6.310e-04	3.339e-03	-0.3354	4.428e-01	9.010e-01	-1.798	2.434e-07	2.590e-06		2
CACNA1A	calcium vo	-1.331	2.231e-03	8.282e-03	-0.1585	4.644e-01	9.040e-01	-1.9387	9.478e-07	8.442e-06		2
CACNA1H	calcium vo	-7.4458	1.232e-08	6.114e-06	NA	NA	NA	-10.6583	2.210e-12	1.242e-10		2
CAVIN2	caveolae a	-3.3207	1.902e-07	2.630e-05	-0.2222	7.507e-01	9.572e-01	-1.5422	1.159e-10	3.476e-09		2
CCL11	C-C motif c	-1.4573	4.055e-03	1.292e-02	NA	NA	NA	-1.0122	3.321e-02	6.683e-02	yes	2
CDH6	cadherin 6	-4.7933	6.465e-10	1.056e-06	-0.3482	8.306e-02	8.962e-01	-6.5198	1.204e-13	1.153e-11		2
CGREF1	cell growth	-1.8075	8.525e-05	8.703e-04	0.1251	8.461e-01	9.732e-01	-4.4081	2.710e-08	3.984e-07	yes	2
CHAC1	ChaC gluta	-1.0917	2.671e-02	5.472e-02	-0.1036	7.968e-01	9.645e-01	-1.4325	4.631e-10	1.198e-08		2
CHL1	cell adhesi	-7.8813	1.047e-04	9.939e-04	-0.0992	5.197e-01	9.132e-01	-5.3858	7.100e-08	9.097e-07	yes	2
CHSY3	chondroitir	-3.5618	6.178e-04	3.292e-03	0.3853	4.120e-01	9.010e-01	-1.0943	2.766e-04	1.130e-03		2
CLGN	calmegin [S	-6.4884	2.118e-06	9.806e-05	0.4888	2.066e-02	8.962e-01	-2.0737	4.515e-06	3.286e-05		2
CMIP	c-Maf indu	-1.2634	8.677e-03	2.288e-02	-0.0099	9.492e-01	9.897e-01	-1.0847	1.169e-09	2.606e-08		2
COL24A1	collagen ty	-4.6072	9.617e-06	2.241e-04	-0.1601	8.273e-01	9.680e-01	-1.0427	9.290e-03	2.266e-02	yes	2
COL8A1	collagen ty	-1.5548	4.614e-06	1.525e-04	-0.2923	6.762e-03	8.962e-01	-1.2797	6.734e-13	4.731e-11	yes	2
COL8A2	collagen ty	-2.5548	1.687e-05	3.244e-04	-0.7055	4.483e-02	8.962e-01	-3.0025	3.571e-13	2.765e-11	yes	2
CPM	carboxype	-2.0361	9.023e-03	2.358e-02	0.2733	5.362e-01	9.165e-01	-1.719	5.958e-05	3.050e-04		2
CPZ	carboxype	-1.782	1.795e-07	2.594e-05	0.1367	8.343e-01	9.700e-01	-3.6728	2.294e-07	2.453e-06	yes	2
CRACD	capping pr	-6.7162	1.663e-05	3.211e-04	NA	NA	NA	-2.1455	2.348e-04	9.790e-04		2
CTHRC1	collagen tr	-2.3866	6.087e-10	1.056e-06	-0.2797	5.531e-02	8.962e-01	-1.0764	2.435e-09	4.933e-08	yes	2
CTSH	cathepsin	-1.1719	3.103e-03	1.051e-02	NA	NA	NA	-1.6725	1.149e-07	1.372e-06		2
CXCL12	C-X-C moti	-3.4924	6.815e-10	1.056e-06	NA	NA	NA	-2.0135	1.707e-06	1.420e-05	yes	2
DCHS1	dachsous c	-1.491	2.639e-02	5.423e-02	-0.7766	2.135e-02	8.962e-01	-3.1003	3.015e-07	3.145e-06		2

DOK6	docking pr	-6.7044	9.494e-06	2.224e-04	0.036	8.920e-01	9.807e-01	-1.2891	1.973e-03	6.116e-03		2
EEPD1	endonucle	-1.0288	9.268e-03	2.405e-02	-0.4084	3.161e-01	8.984e-01	-1.8328	1.631e-08	2.577e-07		2
EID3	EP300 inte	-1.3977	1.791e-03	7.004e-03	-0.1125	8.098e-01	9.676e-01	-1.2604	6.195e-04	2.269e-03		2
ELOVL3	ELOVL fatt	-1.3165	3.007e-03	1.026e-02	NA	NA	NA	-1.0968	6.483e-03	1.672e-02		2
EPDR1	ependymir	-8.2359	9.724e-11	3.193e-07	0.1731	6.139e-01	9.292e-01	-4.4666	2.050e-17	1.893e-14	yes	2
ERFE	erythroferr	-1.5069	1.342e-02	3.201e-02	-0.6771	8.393e-02	8.962e-01	-1.0029	2.909e-08	4.230e-07	yes	2
EYA2	EYA transc	-4.4231	3.657e-06	1.319e-04	0.0977	6.143e-01	9.293e-01	-2.4286	7.214e-08	9.196e-07		2
FAM162B	family with	-1.599	4.859e-03	1.483e-02	-0.0376	9.166e-01	9.840e-01	-3.4484	2.750e-12	1.475e-10		2
FAT1	FAT atypica	-6.4347	8.212e-10	1.056e-06	0.0672	7.770e-01	9.613e-01	-2.1536	1.726e-03	5.450e-03		2
FBN2	fibrillin 2 [S	-1.1053	2.938e-03	1.008e-02	-0.0554	7.568e-01	9.583e-01	-2.3592	1.002e-06	8.846e-06	yes	2
FNBP1L	formin binc	-4.1597	1.514e-06	8.284e-05	0.1506	2.528e-01	8.962e-01	-2.9026	6.412e-06	4.388e-05		2
FOXP2	forkhead b	-4.948	4.360e-05	5.693e-04	-0.1788	5.639e-01	9.212e-01	-3.2868	1.159e-04	5.367e-04		2
GALNT13	polypeptid	-1.976	9.315e-07	6.176e-05	-0.1575	3.940e-01	9.010e-01	-3.24	1.803e-06	1.492e-05		2
GAS1	growth arre	-2.8322	1.945e-04	1.486e-03	-0.3126	2.593e-01	8.962e-01	-1.3266	2.621e-09	5.228e-08		2
GDF6	growth diff	-1.5418	3.231e-05	4.755e-04	NA	NA	NA	-5.3999	3.030e-11	1.103e-09	yes	2
GDF7	growth diff	-1.6581	2.743e-03	9.608e-03	-0.6749	9.438e-04	8.962e-01	-9.0425	1.542e-15	3.437e-13	yes	2
GPNMB	glycoprote	-8.942	8.197e-08	1.787e-05	NA	NA	NA	-4.1109	3.474e-08	4.963e-07		2
GREM2	gremlin 2, I	-1.7732	6.755e-06	1.849e-04	-0.3549	2.195e-01	8.962e-01	-1.3994	3.484e-06	2.631e-05	yes	2
GYPE	glycophori	-1.1593	8.505e-03	2.256e-02	NA	NA	NA	-3.9236	4.777e-05	2.530e-04		2
HAPLN3	hyaluronar	-1.4641	3.284e-05	4.795e-04	0.3864	4.376e-01	9.010e-01	-1.6595	1.840e-07	2.036e-06	yes	2
HAS3	hyaluronar	-2.4564	6.768e-03	1.893e-02	-0.4888	1.259e-01	8.962e-01	-1.4443	3.038e-05	1.717e-04		2
HES4	hes family	-1.1015	1.199e-02	2.939e-02	NA	NA	NA	-2.7438	1.867e-07	2.062e-06		2
HMCN1	hemicentir	-1.0245	1.036e-02	2.618e-02	-0.1574	6.520e-01	9.357e-01	-2.4902	4.358e-04	1.688e-03	yes	2
HOXB3	homeobox	-1.0204	2.940e-02	5.919e-02	0.45	1.180e-01	8.962e-01	-10.8868	3.974e-11	1.374e-09		2
HR	HR lysine c	-1.3207	6.539e-04	3.419e-03	-0.2914	1.655e-01	8.962e-01	-1.75	2.219e-08	3.355e-07		2
HSD17B14	hydroxyste	-7.8421	6.961e-06	1.877e-04	-0.0395	9.213e-01	9.840e-01	-2.367	1.062e-09	2.404e-08		2
HSPB7	heat shock	-2.3808	2.492e-05	4.037e-04	-0.1299	3.464e-01	8.984e-01	-2.696	2.226e-05	1.308e-04		2
IFITM1	interferon i	-2.1416	1.548e-07	2.489e-05	1.1892	1.012e-03	8.962e-01	-2.1934	8.486e-13	5.774e-11		2
IGF2BP1	insulin like	-1.1208	1.220e-02	2.974e-02	-0.0656	7.871e-01	9.620e-01	-2.3311	1.181e-12	7.598e-11		2
IGFBP2	insulin like	-2.672	2.790e-05	4.361e-04	0.3677	4.717e-01	9.063e-01	-2.5611	3.634e-14	4.390e-12	yes	2
IL11	interleukin	-1.5931	1.075e-03	4.866e-03	-1.1503	9.092e-03	8.962e-01	-1.5888	5.284e-08	7.063e-07	yes	3
ITGA11	integrin sul	-1.9317	3.388e-07	3.835e-05	NA	NA	NA	-5.215	6.563e-15	1.075e-12		2
ITGA8	integrin sul	-2.4391	2.606e-03	9.246e-03	NA	NA	NA	-1.8959	4.634e-10	1.198e-08		2

JAM2	junctional	-1.4105	1.171e-02	2.886e-02	-0.0622	6.587e-01	9.373e-01	-1.7724	8.866e-04	3.087e-03		2
KCNH2	potassium	1.0579	8.746e-03	2.303e-02	-1.2664	1.918e-02	8.962e-01	-4.5786	1.492e-11	6.104e-10		2
KCNJ8	potassium	-1.4959	2.209e-02	4.712e-02	1.6638	1.680e-01	8.962e-01	-2.9853	3.471e-09	6.657e-08		2
KCNK1	potassium	-1.0998	9.222e-03	2.397e-02	0.6368	3.250e-01	8.984e-01	-2.5887	2.518e-09	5.054e-08		2
KLHL3	kelch like f	-3.6699	1.787e-05	3.360e-04	-0.1231	6.336e-01	9.335e-01	-1.2558	5.585e-04	2.078e-03		2
KRBOX1	KRAB box c	-2.7367	1.529e-04	1.270e-03	-0.3648	3.332e-01	8.984e-01	-4.9411	1.002e-10	3.063e-09		2
LRATD1	LRAT doma	-5.754	1.649e-06	8.695e-05	-0.3352	5.736e-01	9.229e-01	-2.9813	4.368e-15	8.066e-13		2
LRRC32	leucine ricl	-4.3175	1.121e-04	1.041e-03	0.3592	5.903e-01	9.242e-01	-3.2695	1.902e-10	5.489e-09		2
MCAM	melanoma	-1.982	1.048e-06	6.561e-05	-0.1212	4.514e-01	9.010e-01	-2.1508	3.395e-15	6.650e-13		2
MFAP2	microfibril	-1.3988	1.576e-02	3.629e-02	-0.4255	6.684e-02	8.962e-01	-4.0933	4.018e-12	2.037e-10	yes	2
MFAP4	microfibril	-1.8219	2.230e-07	2.866e-05	0.1328	6.181e-01	9.296e-01	-1.4719	3.610e-10	9.668e-09	yes	2
MFGE8	milk fat glo	-2.1826	2.829e-06	1.159e-04	0.3054	5.466e-01	9.194e-01	-2.2375	6.470e-16	1.885e-13	yes	2
MLF1	myeloid leu	-1.2402	1.703e-02	3.848e-02	0.0737	7.107e-01	9.478e-01	-1.7187	1.151e-04	5.331e-04		2
MME	membrane	-1.1384	1.603e-07	2.545e-05	0.1317	6.426e-01	9.339e-01	-2.7232	4.468e-17	3.208e-14		2
MT3	metallothi	-2.48	1.861e-02	4.120e-02	-1.4736	1.070e-03	8.962e-01	NA	NA	NA		2
MX2	MX dynami	-1.2479	1.001e-02	2.551e-02	0.4404	4.465e-01	9.010e-01	-3.5132	4.403e-12	2.189e-10		2
MYO5B	myosin VB	-1.9613	1.288e-02	3.100e-02	-0.7029	3.638e-02	8.962e-01	-5.2408	1.528e-05	9.426e-05		2
NBL1	NBL1, DAN	-1.0244	7.878e-04	3.909e-03	0.0599	8.138e-01	9.676e-01	-1.7978	4.280e-14	4.929e-12	yes	2
NEFL	neurofilam	-3.9009	9.617e-04	4.477e-03	-0.217	3.946e-01	9.010e-01	-4.2366	1.071e-09	2.420e-08		2
NEFM	neurofilam	-6.426	2.566e-05	4.116e-04	NA	NA	NA	-2.1317	3.132e-06	2.398e-05		2
NFASC	neurofasci	-1.5322	5.934e-03	1.719e-02	0.4152	2.770e-01	8.962e-01	-7.7263	2.002e-15	4.312e-13		2
NLGN1	neuroligin	-4.0317	2.973e-05	4.497e-04	-0.7563	2.674e-02	8.962e-01	-3.5472	6.725e-09	1.206e-07		2
NLGN4X	neuroligin	-8.1975	1.972e-07	2.670e-05	NA	NA	NA	-8.1527	3.509e-07	3.580e-06		2
NOG	noggin [So	-2.4705	4.726e-04	2.727e-03	NA	NA	NA	-1.9247	6.957e-04	2.509e-03	yes	2
NOVA1	NOVA alter	-6.8065	5.036e-05	6.319e-04	NA	NA	NA	-1.4795	3.290e-04	1.315e-03		2
NRCAM	neuronal c	-1.8017	5.852e-03	1.701e-02	0.0772	5.036e-01	9.093e-01	-2.3698	3.380e-08	4.851e-07	yes	2
NXT2	nuclear tra	-2.1275	1.973e-06	9.604e-05	0.1806	5.318e-01	9.160e-01	-2.386	1.391e-12	8.688e-11		2
OSR1	odd-skippe	-1.5936	4.562e-03	1.413e-02	-0.5639	2.975e-01	8.984e-01	-2.0252	6.894e-13	4.791e-11		2
PCBP3	poly(rC) bi	-1.5339	1.035e-04	9.856e-04	NA	NA	NA	-1.0908	1.073e-03	3.639e-03		2
PCDH10	protocadh	-8.7711	2.244e-07	2.866e-05	-0.06	4.523e-01	9.010e-01	-5.0504	2.095e-11	8.109e-10		2
PCDH7	protocadh	-9.1379	9.930e-11	3.193e-07	-0.136	6.997e-01	9.448e-01	-3.1861	8.097e-14	8.579e-12		2
PCDHGB1	protocadh	-1.4657	8.782e-05	8.852e-04	-0.4566	1.537e-02	8.962e-01	-2.0401	6.218e-05	3.162e-04		2
PCDHGB3	protocadh	-2.984	1.938e-04	1.484e-03	0.054	8.682e-01	9.785e-01	-2.347	2.359e-05	1.378e-04		2

PCDHGB5	protocadh	-2.2142	1.140e-06	6.824e-05	0.2982	2.725e-01	8.962e-01	-2.8401	1.685e-13	1.503e-11		2
PCOLCE2	procollage	-1.7898	1.512e-02	3.519e-02	0.7924	1.699e-01	8.962e-01	-2.0752	6.156e-07	5.801e-06	yes	2
PDE1A	phosphodi	-9.0408	9.699e-08	1.883e-05	0.064	7.690e-01	9.612e-01	-3.8156	2.197e-07	2.367e-06		2
PDE3A	phosphodi	-1.8925	1.766e-03	6.922e-03	0.418	4.552e-01	9.010e-01	-4.4233	1.768e-13	1.566e-11		2
PDGFD	platelet de	-1.1553	3.696e-06	1.328e-04	-0.5038	5.077e-03	8.962e-01	-2.0837	1.182e-11	5.009e-10	yes	2
PEAR1	platelet en	-3.9715	5.202e-05	6.421e-04	NA	NA	NA	-1.0278	3.178e-09	6.158e-08		2
PENK	proenkeph	-1.1018	4.836e-02	8.798e-02	-0.1516	6.322e-01	9.331e-01	-2.9268	6.922e-05	3.468e-04	yes	2
PIANP	PILR alpha	-2.4473	1.265e-03	5.423e-03	-0.5722	2.334e-02	8.962e-01	-1.4916	8.935e-05	4.289e-04		2
PLBD1	phospholip	-2.75	2.475e-03	8.896e-03	NA	NA	NA	-4.7454	2.350e-07	2.509e-06		2
POSTN	periostin [S	-3.8432	4.383e-03	1.372e-02	0	1.000e+00	1.000e+00	-5.127	2.069e-14	2.845e-12	yes	2
PROK1	prokinetici	-4.2588	2.391e-02	5.016e-02	NA	NA	NA	-7.8554	6.908e-07	6.443e-06	yes	2
PRSS12	serine prot	-1.5392	4.900e-07	4.600e-05	-0.1489	4.190e-01	9.010e-01	-1.7103	9.609e-10	2.226e-08	yes	2
PTGER3	prostaglan	-6.369	1.273e-08	6.114e-06	0.4814	4.113e-01	9.010e-01	-7.3744	2.226e-14	2.991e-12		2
PTPRD	protein tyr	-6.7344	8.595e-06	2.119e-04	0.1345	4.438e-01	9.010e-01	-7.6325	2.152e-06	1.729e-05		2
PYGM	glycogen p	-2.6753	1.040e-04	9.898e-04	-0.3658	2.691e-01	8.962e-01	-1.9701	1.924e-04	8.247e-04		2
RAI2	retinoic ac	-4.7094	6.895e-05	7.631e-04	NA	NA	NA	-3.9684	4.295e-06	3.155e-05		2
RAMP1	receptor ac	-3.3449	2.365e-04	1.694e-03	0.852	9.267e-02	8.962e-01	-2.2842	3.297e-13	2.567e-11		2
RASGRP2	RAS guany	-1.3786	1.083e-02	2.712e-02	-0.1263	8.367e-01	9.708e-01	-4.2246	1.961e-12	1.136e-10		2
RBM24	RNA bindir	-1.0248	8.231e-04	4.019e-03	0.4425	2.895e-01	8.984e-01	-1.5714	3.808e-09	7.228e-08		2
RGCC	regulator o	-1.8918	4.516e-04	2.643e-03	0.1598	7.113e-01	9.478e-01	-1.3926	8.924e-11	2.773e-09		2
RGN	regucalcin	-1.4883	2.620e-02	5.392e-02	-0.0499	8.418e-01	9.718e-01	-2.9601	3.713e-07	3.738e-06		2
RNF150	ring finger p	-1.3632	1.058e-03	4.813e-03	0.1855	3.510e-01	8.984e-01	-4.9989	5.272e-12	2.552e-10		2
RNF182	ring finger p	-1.4374	4.024e-02	7.592e-02	NA	NA	NA	-5.9526	1.279e-06	1.094e-05		2
RORB	RAR relate	-3.7386	9.224e-06	2.194e-04	-0.0984	8.236e-01	9.676e-01	-2.007	8.214e-08	1.021e-06		2
RUNX1T1	RUNX1 par	-2.2221	1.285e-04	1.139e-03	NA	NA	NA	-5.7133	9.807e-09	1.668e-07		2
SAMD5	sterile alph	-3.8338	6.557e-07	5.174e-05	0.1111	6.998e-01	9.448e-01	-2.5295	2.380e-07	2.536e-06		2
SATB1	SATB home	-3.5472	5.016e-07	4.644e-05	0.2708	1.747e-01	8.962e-01	-1.5301	3.127e-04	1.257e-03		2
SERPING1	serpin fam	-1.1153	5.215e-06	1.620e-04	-0.0704	7.531e-01	9.577e-01	-1.9087	9.025e-14	9.260e-12	yes	2
SERTM2	serine rich	-9.5601	1.290e-08	6.114e-06	NA	NA	NA	-8.5018	8.142e-08	1.017e-06		2
SGCE	sarcoglyca	-2.1138	9.501e-08	1.883e-05	-0.0251	8.693e-01	9.788e-01	-3.9111	1.915e-16	8.059e-14		2
SHISAL1	shisa like 1	-3.1899	1.783e-05	3.358e-04	-0.9454	1.679e-02	8.962e-01	-1.0884	1.767e-04	7.689e-04		2
SIGIRR	single Ig ar	-2.3796	3.177e-05	4.713e-04	-0.1164	7.088e-01	9.471e-01	-1.8023	3.936e-07	3.938e-06		2
SLC27A6	solute carr	-4.919	3.169e-07	3.672e-05	0.4201	9.185e-02	8.962e-01	-1.7891	6.428e-11	2.083e-09		2

SORBS2	sorbin and	-4.6686	2.432e-04	1.726e-03	-0.6473	7.975e-02	8.962e-01	-5.1101	2.584e-10	7.198e-09		2
SOX15	SRY-box tra	-1.4269	1.110e-02	2.765e-02	-0.983	6.184e-02	8.962e-01	-1.2841	1.940e-03	6.021e-03		2
SPEG	striated mu	-1.4115	3.549e-05	5.029e-04	0.2315	2.902e-01	8.984e-01	-2.2318	2.516e-03	7.492e-03		2
SPESP1	sperm equ	-6.1831	1.199e-05	2.578e-04	NA	NA	NA	-3.0573	1.903e-04	8.176e-04	yes	2
SPHK1	sphingosin	-1.7111	7.071e-05	7.740e-04	0.3468	4.840e-01	9.066e-01	-1.1647	3.092e-05	1.739e-04		2
ST6GALNA	ST6 N-acet	-2.2516	9.120e-03	2.375e-02	-0.3019	6.994e-01	9.448e-01	-4.3628	7.044e-11	2.254e-09		2
STAC	SH3 and cy	-8.0861	9.849e-05	9.536e-04	0.2638	4.925e-01	9.075e-01	-2.4958	4.582e-05	2.446e-04		2
SULF1	sulfatase 1	-2.6045	1.863e-02	4.123e-02	0.9916	3.196e-02	8.962e-01	-3.5837	5.342e-12	2.567e-10		2
SYT1	synaptotag	-2.9561	2.155e-04	1.589e-03	-0.1102	4.445e-01	9.010e-01	-4.9476	2.271e-12	1.271e-10		2
SYTL5	synaptotag	-2.5316	1.482e-03	6.083e-03	-0.2081	5.992e-01	9.243e-01	-4.3428	3.400e-09	6.540e-08		2
TCEAL7	transcripti	-1.4261	2.398e-03	8.711e-03	0.0488	8.113e-01	9.676e-01	-1.426	2.794e-05	1.598e-04		2
THY1	Thy-1 cell s	-1.4333	1.507e-06	8.284e-05	-0.0131	9.617e-01	9.907e-01	-1.7426	8.365e-14	8.720e-12		2
TINAGL1	tubulointer	-1.8093	1.118e-03	4.997e-03	NA	NA	NA	-5.7691	1.225e-09	2.703e-08	yes	2
TLE6	TLE family	-1.3139	2.766e-02	5.623e-02	-0.2779	3.687e-01	8.984e-01	-1.5505	2.088e-03	6.400e-03		2
TMEFF2	transmeml	-7.9406	1.497e-04	1.253e-03	0.1631	5.504e-01	9.196e-01	-2.4371	2.710e-05	1.557e-04	yes	2
TMEM204	transmeml	-4.1903	5.169e-04	2.896e-03	0.0591	8.116e-01	9.676e-01	-1.05	5.887e-08	7.718e-07		2
TMEM59L	transmeml	-3.5514	1.731e-06	9.052e-05	0.1055	7.859e-01	9.620e-01	-1.3594	2.254e-04	9.451e-04		2
TMEM74B	transmeml	-4.8649	1.247e-03	5.376e-03	-1.0906	2.211e-02	8.962e-01	NA	NA	NA		2
TPD52L1	TPD52 like	-1.7063	2.998e-03	1.024e-02	0.4965	4.942e-01	9.076e-01	-1.5197	4.343e-03	1.186e-02		2
USP32P1	ubiquitin s	-1.951	3.459e-04	2.195e-03	0.0265	9.339e-01	9.868e-01	-2.5384	3.746e-05	2.051e-04		2
VIPR2	vasoactive	-2.1445	5.140e-03	1.546e-02	-0.0859	7.330e-01	9.546e-01	-2.9036	4.033e-12	2.037e-10		2
WDR86	WD repeat	-2.7886	2.869e-05	4.435e-04	NA	NA	NA	-2.0284	1.513e-12	9.311e-11		2
WNT2	Wnt family	-1.1217	8.569e-03	2.267e-02	-0.3473	4.075e-01	9.010e-01	-1.1933	4.539e-06	3.296e-05	yes	2
ZNF474	zinc finger	-1.1058	4.168e-02	7.805e-02	NA	NA	NA	-1.0292	1.227e-02	2.871e-02		2
