# Supplementary material for: Primary BPH Stromal Cell Cultures Exhibit Heightened Androgen‐Responsiveness
Source: Prostate. 2026 Aug 30;86(15):1508–17. doi: 10.1002/pros.70239 (PMC13656324; doi:10.1002/pros.70239)
Supplement: Supplementary file 5 [file PROS-86-1508-s005.pdf]

TP18-S7

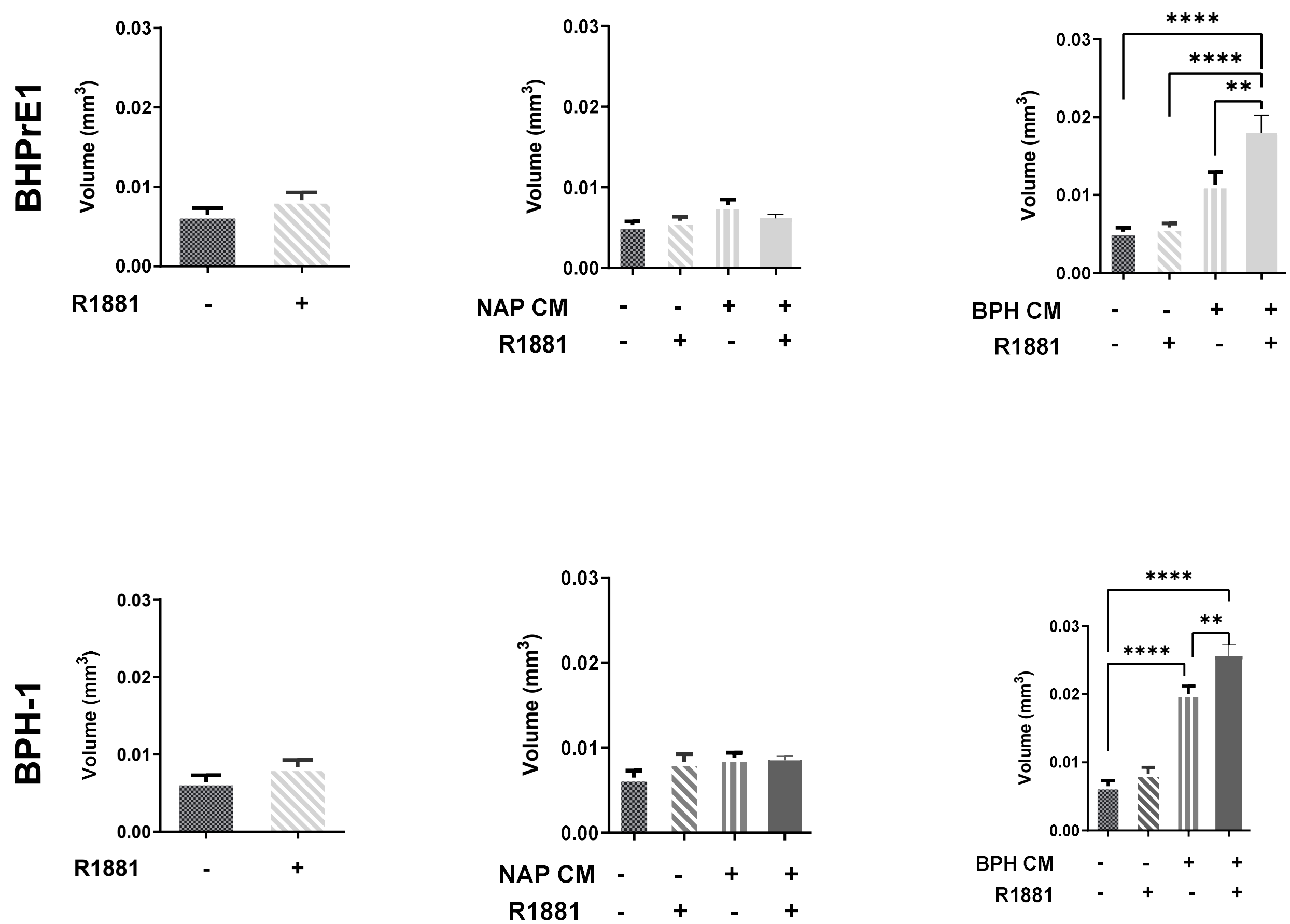

Supplemental Fig. 1. BPH stromal cells mediate androgen-induced epithelial proliferation in 3D culture in BHPrE1 (Upper panel) and BPH-1 (Lower panel) as described in Fig. 6. The BPH and NAP stromal cells were derived from the same patient (TP18-S7).
