# Supplementary material for: Primary BPH Stromal Cell Cultures Exhibit Heightened Androgen‐Responsiveness
Source: Prostate. 2026 Aug 30;86(15):1508–17. doi: 10.1002/pros.70239 (PMC13656324; doi:10.1002/pros.70239)
Supplement: Supplementary file 6 [file PROS-86-1508-s006.pdf]

Up regulated genes in BPH

| hgnc_symb | descriptio | S7_logFC | S7_PValue | S7_FDR | S279_logF | S279_PVal | S279_FDR | S558_logF | S558_PVal | S558_FDR | secreted p | patients_with_DEG |
|---|---|---|---|---|---|---|---|---|---|---|---|---|
| A2M | alpha-2-ma | 4.8503 | 1.498e-04 | 1.253e-03 | NA | NA | NA | 1.0044 | 1.380e-06 | 1.170e-05 | yes | 2 |
| AKR1B10 | aldo-keto r | -0.5189 | 4.917e-02 | 8.913e-02 | 1.7058 | 1.868e-03 | 8.962e-01 | 2.008 | 7.444e-07 | 6.888e-06 | yes | 2 |
| AKR1C1 | aldo-keto r | -0.6125 | 2.215e-01 | 3.051e-01 | 1.4258 | 4.808e-02 | 8.962e-01 | 2.693 | 7.155e-17 | 4.625e-14 | | 2 |
| ANK3 | ankyrin 3 [S | 2.035 | 5.165e-06 | 1.617e-04 | 0.1348 | 6.591e-01 | 9.373e-01 | 3.3666 | 4.101e-07 | 4.078e-06 | | 2 |
| ARHGAP28 | Rho GTPas | 1.0224 | 1.888e-06 | 9.449e-05 | -0.4571 | 4.607e-01 | 9.026e-01 | 3.1994 | 1.407e-12 | 8.746e-11 | | 2 |
| BCL2L11 | BCL2 like 1 | -1.9954 | 2.243e-04 | 1.633e-03 | 2.2651 | 4.511e-02 | 8.962e-01 | 1.4626 | 7.065e-04 | 2.544e-03 | | 2 |
| C3 | compleme | 1.8659 | 2.960e-05 | 4.490e-04 | -0.569 | 4.734e-03 | 8.962e-01 | 1.7342 | 3.002e-09 | 5.862e-08 | yes | 2 |
| C4orf33 | chromosor | 1.0403 | 2.199e-03 | 8.204e-03 | 0.2257 | 6.173e-01 | 9.296e-01 | 1.2279 | 7.781e-05 | 3.810e-04 | | 2 |
| CCND2 | cyclin D2 [S | 1.3049 | 4.852e-03 | 1.482e-02 | 0.0881 | 5.362e-01 | 9.165e-01 | 1.6073 | 4.028e-11 | 1.388e-09 | | 2 |
| CDC20 | cell divisio | 1.0874 | 2.483e-02 | 5.167e-02 | -0.8395 | 2.231e-01 | 8.962e-01 | 1.0946 | 8.966e-05 | 4.302e-04 | | 2 |
| CDK15 | cyclin depe | 1.0798 | 7.787e-03 | 2.106e-02 | -0.1216 | 7.824e-01 | 9.613e-01 | 1.8209 | 2.686e-11 | 1.009e-09 | | 2 |
| CDK18 | cyclin depe | 1.9003 | 2.896e-02 | 5.848e-02 | -1.4286 | 2.110e-04 | 8.170e-01 | 2.1629 | 3.962e-05 | 2.156e-04 | | 2 |
| CHD7 | chromodor | 1.7372 | 8.111e-04 | 3.982e-03 | 1.0165 | 4.521e-02 | 8.962e-01 | NA | NA | NA | | 2 |
| CIT | citron rho-i | 1.3014 | 6.514e-06 | 1.798e-04 | -0.1585 | 6.814e-01 | 9.412e-01 | 1.2831 | 5.465e-06 | 3.852e-05 | | 2 |
| COLEC10 | collectin su | 3.1163 | 2.275e-05 | 3.831e-04 | -0.3964 | 1.876e-01 | 8.962e-01 | 1.8006 | 6.863e-12 | 3.130e-10 | yes | 2 |
| CYP2S1 | cytochrom | 1.3759 | 3.615e-05 | 5.064e-04 | 0.5583 | 3.092e-01 | 8.984e-01 | 2.6829 | 3.722e-12 | 1.924e-10 | | 2 |
| DDO | D-aspartat | 1.7433 | 2.495e-03 | 8.945e-03 | 0.2594 | 3.717e-01 | 8.984e-01 | 2.894 | 4.632e-07 | 4.516e-06 | | 2 |
| DNHD1 | dynein hea | -0.4577 | 1.438e-01 | 2.140e-01 | 1.4968 | 3.901e-02 | 8.962e-01 | 1.2949 | 1.675e-06 | 1.394e-05 | | 2 |
| DSP | desmoplak | 4.322 | 7.109e-08 | 1.669e-05 | 1.1282 | 2.584e-02 | 8.962e-01 | -2.3148 | 6.908e-03 | 1.765e-02 | | 2 |
| DTX4 | deltex E3 u | 1.0737 | 3.116e-05 | 4.639e-04 | -0.3366 | 4.310e-01 | 9.010e-01 | 1.1987 | 1.710e-11 | 6.864e-10 | | 2 |
| EEF1A2 | eukaryotic | 2.0516 | 6.482e-06 | 1.797e-04 | 0.4771 | 4.513e-01 | 9.010e-01 | 2.27 | 9.002e-06 | 5.922e-05 | | 2 |
| ELL3 | elongation | 1.1425 | 3.005e-02 | 6.021e-02 | NA | NA | NA | 1.1121 | 1.696e-02 | 3.791e-02 | | 2 |
| EMILIN2 | elastin mic | 1.0419 | 3.151e-03 | 1.062e-02 | -0.1607 | 4.348e-01 | 9.010e-01 | 3.2283 | 2.736e-15 | 5.744e-13 | yes | 2 |
| ENPP4 | ectonuclec | 1.5825 | 6.661e-03 | 1.872e-02 | 0.5607 | 4.591e-01 | 9.026e-01 | 1.4926 | 3.505e-03 | 9.890e-03 | | 2 |
| FBXW10B | F-box and V | -0.4598 | 4.237e-01 | 5.165e-01 | 1.1151 | 4.398e-02 | 8.962e-01 | 2.5653 | 8.693e-07 | 7.886e-06 | | 2 |
| FLRT2 | fibronectin | 1.482 | 5.383e-03 | 1.600e-02 | -0.1763 | 2.973e-01 | 8.984e-01 | 1.3734 | 1.305e-07 | 1.525e-06 | | 2 |
| FRK | fyn related | 2.2888 | 8.713e-07 | 6.091e-05 | 0.1652 | 6.878e-01 | 9.417e-01 | 1.5422 | 1.785e-07 | 1.984e-06 | | 2 |
| GAL | galanin and | 3.7131 | 8.699e-08 | 1.865e-05 | 1.2761 | 1.677e-03 | 8.962e-01 | 3.6759 | 7.330e-08 | 9.289e-07 | yes | 3 |
| GCOM1 | GCOM1, M | 2.6424 | 1.643e-03 | 6.563e-03 | 0.4242 | 5.917e-01 | 9.243e-01 | 1.2142 | 1.113e-02 | 2.650e-02 | | 2 |
| GRK3 | G protein-c | 1.3333 | 2.328e-03 | 8.529e-03 | 0.6072 | 1.940e-01 | 8.962e-01 | 1.7619 | 5.715e-06 | 4.001e-05 | | 2 |
| HHIPL2 | HHIP like 2 | 2.088 | 1.713e-03 | 6.769e-03 | NA | NA | NA | 1.3951 | 2.683e-04 | 1.099e-03 | yes | 2 |

| | | | | | | | | | | | | |
|---|---|---|---|---|---|---|---|---|---|---|---|---|
| HIF3A | hypoxia inc | 1.5984 | 5.246e-04 | 2.932e-03 | -0.1241 | 7.887e-01 | 9.620e-01 | 1.7469 | 9.875e-08 | 1.198e-06 | | 2 |
| ICAM4 | intercellula | 1.1778 | 1.661e-03 | 6.624e-03 | NA | NA | NA | 4.122 | 4.691e-08 | 6.389e-07 | | 2 |
| ICAM5 | intercellula | 0.1268 | 3.034e-01 | 3.948e-01 | 1.5339 | 1.808e-02 | 8.962e-01 | 2.5608 | 2.095e-10 | 5.952e-09 | | 2 |
| IFI6 | interferon a | 1.073 | 1.583e-04 | 1.293e-03 | 1.0777 | 2.890e-04 | 8.170e-01 | -1.7446 | 8.450e-06 | 5.595e-05 | | 2 |
| IRX3 | iroquois hc | 2.3022 | 2.143e-04 | 1.583e-03 | NA | NA | NA | 3.0655 | 1.394e-07 | 1.618e-06 | | 2 |
| ITPKB | inositol-tris | 1.318 | 2.982e-04 | 1.987e-03 | -0.1416 | 4.498e-01 | 9.010e-01 | 1.2368 | 1.761e-06 | 1.461e-05 | | 2 |
| KCTD14 | potassium | 1.8464 | 4.051e-04 | 2.450e-03 | NA | NA | NA | 2.4422 | 1.143e-05 | 7.297e-05 | | 2 |
| LGALSL | galectin lik | 2.1479 | 6.261e-04 | 3.321e-03 | NA | NA | NA | 1.2368 | 1.538e-04 | 6.818e-04 | | 2 |
| LRRC17 | leucine ricl | 1.4976 | 9.802e-03 | 2.509e-02 | -0.1442 | 3.761e-01 | 8.997e-01 | 1.8779 | 4.625e-13 | 3.416e-11 | yes | 2 |
| LRRC2 | leucine ricl | 1.5894 | 1.061e-04 | 1.002e-03 | NA | NA | NA | 1.7905 | 1.462e-04 | 6.528e-04 | | 2 |
| LTK | leukocyte r | 2.4483 | 9.492e-04 | 4.438e-03 | -0.1637 | 7.156e-01 | 9.490e-01 | 3.1218 | 7.042e-07 | 6.544e-06 | | 2 |
| MAOA | monoamin | 1.528 | 8.331e-03 | 2.218e-02 | 0.2674 | 1.474e-01 | 8.962e-01 | 1.188 | 7.611e-11 | 2.406e-09 | | 2 |
| MMP28 | matrix met | 1.7198 | 5.054e-05 | 6.332e-04 | 0.0644 | 8.240e-01 | 9.676e-01 | 1.9628 | 6.605e-09 | 1.189e-07 | yes | 2 |
| NACAD | NAC alpha | 1.415 | 6.245e-06 | 1.771e-04 | -0.2373 | 3.004e-01 | 8.984e-01 | 1.0156 | 5.840e-07 | 5.547e-06 | | 2 |
| OR5AS1 | olfactory re | 1.0391 | 2.424e-02 | 5.073e-02 | -0.1515 | 6.427e-01 | 9.339e-01 | 1.2485 | 2.070e-02 | 4.482e-02 | | 2 |
| PDE4C | phosphodi | -1.6317 | 6.126e-03 | 1.757e-02 | 1.1278 | 7.231e-03 | 8.962e-01 | 1.4839 | 2.785e-05 | 1.594e-04 | | 2 |
| PGF | placental g | 3.2833 | 1.260e-05 | 2.662e-04 | NA | NA | NA | 6.6783 | 1.749e-11 | 6.998e-10 | yes | 2 |
| PIWIL3 | piwi like RI | 1.8847 | 5.843e-05 | 6.863e-04 | -0.024 | 9.696e-01 | 9.927e-01 | 1.7342 | 1.206e-03 | 4.000e-03 | | 2 |
| PLPPR3 | phospholip | 2.4439 | 2.188e-04 | 1.608e-03 | 0.2408 | 7.714e-01 | 9.612e-01 | 1.1972 | 5.078e-06 | 3.617e-05 | | 2 |
| PLXDC2 | plexin dom | 5.2537 | 1.289e-11 | 1.658e-07 | -0.1877 | 4.605e-01 | 9.026e-01 | 3.8949 | 2.977e-17 | 2.264e-14 | | 2 |
| PLXNA2 | plexin A2 [S | 1.3363 | 4.032e-03 | 1.286e-02 | NA | NA | NA | 1.4613 | 3.767e-05 | 2.062e-04 | | 2 |
| PRXL2A | peroxiredo | 1.3918 | 1.585e-04 | 1.294e-03 | 0.2965 | 5.636e-01 | 9.212e-01 | 1.4554 | 4.112e-12 | 2.068e-10 | yes | 2 |
| PTPRF | protein tyro | 1.4047 | 1.716e-07 | 2.559e-05 | -0.146 | 4.307e-01 | 9.010e-01 | 1.78 | 5.090e-15 | 9.139e-13 | | 2 |
| RAP1GAP2 | RAP1 GTPa | 3.2913 | 6.656e-07 | 5.188e-05 | 0.0306 | 7.715e-01 | 9.612e-01 | 1.2549 | 7.051e-07 | 6.548e-06 | | 2 |
| RASSF5 | Ras associ | 1.1895 | 2.437e-03 | 8.801e-03 | NA | NA | NA | 1.7262 | 1.005e-04 | 4.754e-04 | | 2 |
| SAA1 | serum amy | 4.1212 | 4.861e-10 | 1.056e-06 | 0.6148 | 3.812e-01 | 9.008e-01 | 1.3487 | 1.358e-07 | 1.582e-06 | yes | 2 |
| SAA2 | serum amy | 3.6577 | 3.056e-06 | 1.191e-04 | 1.4669 | 2.987e-01 | 8.984e-01 | 2.2553 | 3.310e-05 | 1.842e-04 | yes | 2 |
| SBSPON | somatome | 2.5948 | 8.219e-05 | 8.520e-04 | NA | NA | NA | 3.5232 | 3.649e-13 | 2.808e-11 | yes | 2 |
| SCUBE1 | signal pept | 1.1618 | 1.202e-02 | 2.942e-02 | NA | NA | NA | 4.0287 | 3.548e-11 | 1.246e-09 | yes | 2 |
| SDK2 | sidekick ce | 3.4064 | 2.929e-05 | 4.463e-04 | -0.6976 | 1.355e-01 | 8.962e-01 | 2.7536 | 1.513e-07 | 1.740e-06 | | 2 |
| SEMA4D | semaphori | 1.1136 | 8.482e-04 | 4.089e-03 | 0.2855 | 4.100e-01 | 9.010e-01 | 1.239 | 1.072e-08 | 1.798e-07 | | 2 |
| STAP2 | signal tran | 1.5671 | 2.289e-04 | 1.655e-03 | -0.044 | 8.670e-01 | 9.785e-01 | 1.5115 | 1.613e-05 | 9.846e-05 | | 2 |
| STOX2 | storkhead | 1.0549 | 4.599e-03 | 1.422e-02 | 0.1259 | 9.055e-01 | 9.815e-01 | 1.115 | 4.143e-06 | 3.066e-05 | | 2 |

| | | | | | | | | | | | | |
|---|---|---|---|---|---|---|---|---|---|---|---|---|
| STRA6 | signaling re | 2.6441 | 4.303e-07 | 4.272e-05 | NA | NA | NA | 2.9174 | 5.226e-14 | 5.774e-12 | | 2 |
| TCIM | transcriptio | 1.2785 | 2.050e-02 | 4.455e-02 | NA | NA | NA | 2.2189 | 1.907e-09 | 3.956e-08 | | 2 |
| TMEM229E | transmeml | 1.1617 | 2.767e-03 | 9.678e-03 | -0.1852 | 5.382e-01 | 9.167e-01 | 2.1099 | 2.850e-04 | 1.160e-03 | | 2 |
| TNFRSF11I | TNF recept | 1.482 | 8.146e-03 | 2.180e-02 | -1.0552 | 1.171e-02 | 8.962e-01 | 2.0042 | 4.308e-14 | 4.929e-12 | yes | 2 |
| TNFRSF1B | TNF recept | 1.234 | 1.013e-05 | 2.311e-04 | NA | NA | NA | 1.5858 | 3.962e-11 | 1.373e-09 | yes | 2 |
| TRBC2 | T cell recep | 1.3156 | 6.337e-03 | 1.806e-02 | NA | NA | NA | 1.3954 | 6.731e-03 | 1.725e-02 | | 2 |
| VGF | VGF nerve | 1.2452 | 3.079e-02 | 6.144e-02 | 1.6862 | 2.235e-02 | 8.962e-01 | 1.6615 | 4.715e-03 | 1.272e-02 | yes | 3 |
| ZFP92 | ZFP92 zinc | 1.2949 | 3.267e-03 | 1.093e-02 | NA | NA | NA | 2.1439 | 4.474e-03 | 1.216e-02 | | 2 |
